# Carbon nanotube electrodes for retinal implants: A study of structural and functional integration over time

**DOI:** 10.1016/j.biomaterials.2016.10.018

**Published:** 2017-01

**Authors:** Cyril G. Eleftheriou, Jonas B. Zimmermann, Henrik D. Kjeldsen, Moshe David-Pur, Yael Hanein, Evelyne Sernagor

**Affiliations:** a Institute of Neuroscience, Faculty of Medical Sciences, Newcastle University, Framlington Place, Newcastle-upon-Tyne, NE2 4HH, United Kingdom; b School of Electrical Engineering, Tel-Aviv University, Ramat-Aviv, Tel-Aviv, 69978, Israel

**Keywords:** Retina, Prosthesis, Carbon nanotubes (CNTs), Multi electrode array (MEA), Inner limiting membrane (ILM), Glia

## Abstract

The choice of electrode material is of paramount importance in neural prosthetic devices. Electrodes must be biocompatible yet able to sustain repetitive current injections in a highly corrosive environment. We explored the suitability of carbon nanotube (CNT) electrodes to stimulate retinal ganglion cells (RGCs) in a mouse model of outer retinal degeneration. We investigated morphological changes at the bio-hybrid interface and changes in RGC responses to electrical stimulation following prolonged *in vitro* coupling to CNT electrodes.

We observed gradual remodelling of the inner retina to incorporate CNT assemblies. Electrophysiological recordings demonstrate a progressive increase in coupling between RGCs and the CNT electrodes over three days, characterized by a gradual decrease in stimulation thresholds and increase in cellular recruitment. These results provide novel evidence for time-dependent formation of viable bio-hybrids between CNTs and the retina, demonstrating that CNTs are a promising material for inclusion in retinal prosthetic devices.

## 1 Introduction

Retinal prostheses aim to restore a degree of vision in patients with photoreceptor degeneration. The principle is either to take advantage of the surviving retinal circuitry or to target retinal ganglion cells (RGCs) directly, the output channels from the eye to the brain, encoding visual scenes into spike trains which are then transmitted to central visual targets via the optic nerve [1], [2]. Epi-retinal prostheses consist of micro-electrode arrays (MEAs) apposed to the vitreal side of the retina, providing direct electrical stimulation to the RGC layer [3]. Individual electrodes have to deliver electrical pulses strong enough to elicit action potentials in surrounding RGCs without damaging the electrode material or the target tissue [4].

As the main interface between the prosthetic device and the tissue, electrodes are critically important components of any neuro-prosthetic system. For epi-retinal prosthetic devices, maximal resolution would ideally involve stimulation of individual RGCs, requiring electrode sizes to match those of their target neurons. However, small electrodes require higher charge densities to provide sufficient power to drive cells above firing threshold. If the charge density is too high, it can damage the tissue or the electrode, rendering the system unusable in the long term. Hence, it is important to estimate safe charge density limits based on electrode and tissue properties, allowing the system to function safely over prolonged periods. An optimal epi-retinal stimulation system would thus include very small, capacitive electrodes, located in close proximity to the RGC layer, requiring low amounts of current to depolarise RGCs to threshold. Small electrode dimensions guarantee stimulation localization and capacitive electrodes help avoiding direct charge injection into the tissue and undesired Faradaic reaction.

As charge density is intrinsically related to the effective surface area of the electrode, the geometry of stimulating electrodes strongly affects their charge density limit. As such, materials with large effective surface areas are ideal for efficient stimulation. Materials such as titanium nitride (TiN), iridium oxide (IrOx) and platinum grey are considered as gold standards for neural prosthetics and used respectively by the three main retinal prosthetic projects [5], [6], [7]. Carbon nanotubes (CNTs) have attracted much attention since their emergence in the field of bioengineering [8] due to their outstanding electrical [9], [10], chemical and mechanical properties. Their high surface area [11], remarkable tensile strength [12], biocompatibility [13] and high conductivity [14] make them an alluring candidate material to use in neural prosthetic electrodes [15], [16], [17].

Neurodegeneration is characterized by strong glial proliferation, and rejection of epi-retinal electrodes by glial cells can potentially widen the gap between electrodes and their target neurons, thus increasing the amount of charge required to elicit action potentials. The glial population of the retina, consisting of microglia (scattered throughout the inner retinal layers), astrocytes (horizontal syncytium at the nerve fibre layer) and Müller cells (spanning the retina transversely) maintain homeostasis [18], [19], provide immunological protection [20], [21] and structural support [22], [23]. Macroglia (astrocytes and Müller Cells) are also the source of the inner limiting membrane (ILM), a basement membrane providing physical and electrostatic barrier between the vitreous and the retina [24]. Similar to other basement membranes, the ILM is composed of three layers [25]: the *lamina rara interna* (adjacent to glial cell endfeet), the *lamina densa* and the *lamina rara externa* (contiguous with the vitreous humour). These layers have the typical molecular constitution found in basement membranes with laminins, collagen, and other heparan sulfate proteoglycans [26], [27], [28], [29]. Integration of epi-retinal electrodes into the ILM is essential to reduce the distance between stimulating electrodes and target neurons.

Understanding the interactions between stimulating electrodes and the complex micro-environment at the vitreo-retinal interface is thus an important step for the successful design of efficient epi-retinal prosthetic devices. In this study, we investigated the response of RGCs, macroglial cells and the ILM of dystrophic retinas (here, we used the Crx knockout mouse model of Leber congenital amaurosis [30], [31]) to interfacing with CNT electrodes over up to three days *in vitro*. During this incubation period, we observed a substantial increase in the coupling between RGCs and CNT electrodes, reflected both by electrophysiological and anatomical changes. Signals exhibited a gradual improvement in signal-to-noise ratio and decrease in stimulation thresholds. At the same time, CNT structures became gradually integrated into the ILM, reducing the distance between electrodes and RGCs.

## 2 Materials and methods

### 2.1 Device fabrication

**Fig. 1:**
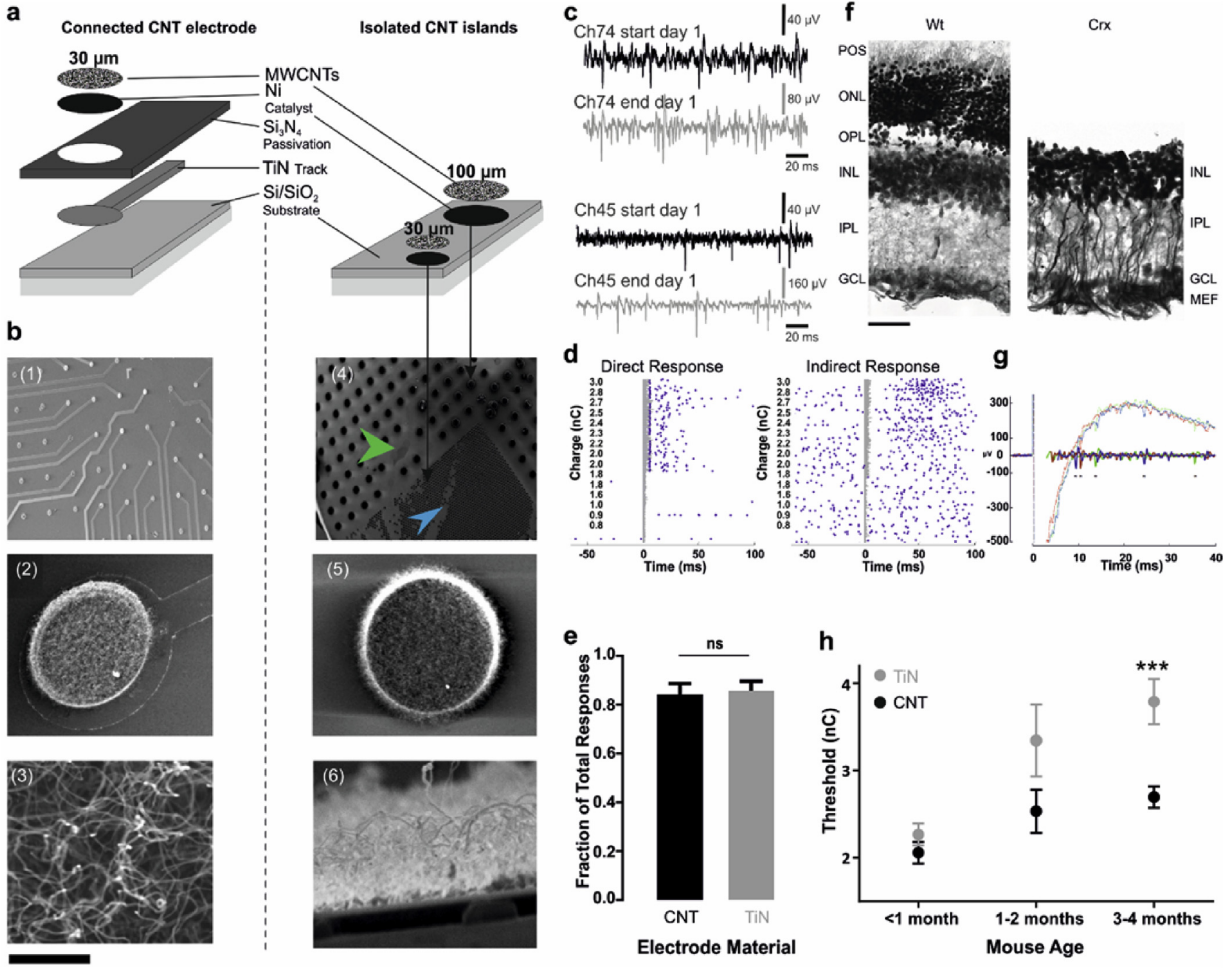
**Carbon Nanotube (CNT) electrodes require lower activation thresholds in advanced stages of retinal degeneration and record larger spikes than Titanium Nitride (TiN) electrodes. (a)** Manufacturing CNT Multielectrode Arrays (MEAs; left) begins with a Si/SiO_2_ substrate on which is deposited conductive TiN and passivating Si_3_N_4_. A 30 μm hole is etched into the passivation layer before depositing Ni and growing CNTs by CVD, yielding electrically connected CNT electrodes. Isolated detachable CNT islands (right) are manufactured by patterning Ni directly onto a Si/SiO_2_ substrate and growing CNTs by CVD, yielding concentric lattices of detachable 100 μm and 30 μm CNT islands. **(b)** Scanning electron micrographs (SEM) at increasing magnification revealing the surface of a CNT MEA (30 μm electrodes with 200 μm pitch) **(b1-3)** and loose CNT islands **(b4-6)**, highlighting the large surface area of entangled CNTs. Arrowheads in b4 indicate areas where lose 100 μm and 30 μm islands have been displaced. Scale bars are **b1** 500 μm, **b2** 60 μm, **b3** 400 nm, **b4** 1 mm, **b5** 6 μm, **b6** 2 μm. **(c)** Example raw trace displaying spike sizes increasing over the course of 8 h recorded on two CNT electrodes. **(d)** Raster plots displaying examples of direct (left) and indirect (right) responses recorded on a CNT electrode. **(e)** Average fraction of direct responses per retina for experiments performed with both TiN and CNT electrodes. Mann Whitney test, p = 0.9609, N = 11 retinas on CNT MEAs and 6 retinas on TiN MEAs. **(f)** Wild type and Cone-Rod Homeobox knockout (Crx−/−) retinal sections (from 3 months old mice) stained for Glial Fibrillary Acidic Protein (GFAP) with a Nissl counterstain, highlighting the lack of an outer retina and sealing of the inner retina by hypertrophic Müller cells. Scale bars: 50 μm. **(g)** Typical example of the stimulation artefact. Three traces of raw data (dashed lines) and the corresponding cleaned traces (using the SALPA correction algorithm [38], bold lines) are shown. Due to saturation of the amplifier, the first few milliseconds of the recording are lost. **(h)** Longitudinal changes in charge thresholds (average ± SEM) for CNT and TiN electrodes in Crx −/− retinas during the first four postnatal months. N (CNT <1, 1–2 and 3–4 months) = 54, 6, 63. N (TiN < 1, 1–2 and 3–4 months) = 48, 14, 39. ***- p = 0.0002, Mann Whitney test.

We fabricated both MWCNT (Multi-walled CNT) MEAs and isolated MWCNT islands (Fig. 1a) to investigate respectively the electrophysiological and morphological impact of CNT electrodes interfaced with the inner retina. Our CNT assemblies displayed a high surface area characteristic of unaligned CNTs grown by chemical vapour deposition (CVD, Fig. 1b). Active MEAs were used to stimulate cells and to record electrophysiological signals from the RGC layer in intact, live tissue. Passive MEAs were used to study the structural integration of CNT islands (initially just loosely attached to a silicon/silicon dioxide (Si/SiO_2_) substrate) into retinal tissue.

The fabrication process for electrically connected CNT MEAs has been described elsewhere [17], and isolated CNT islands were fabricated using a similar process, but involving fewer steps [32]. Briefly, Ni was patterned on Si/SiO_2_ substrates by photo-lithography, and then used as a catalyst to grow CNTs by CVD. For active MEAs, the CNT islands had a 30 μm diameter and a 200 μm pitch (30/200). Both processes require nickel, a toxic metal, to be used as a catalyst for CNT growth. Nickel is effectively embedded in the CNTs and has no adverse effects on neuronal cells, as validated by long term culturing experiments and energy-dispersive x-ray spectroscopy testing [32]. Long-term stability of our CNT electrodes was previously tested extensively in cell cultures [33].

Passive MEAs had two types of islands. 30 μm islands were organised in a central orthogonal lattice and had a 50 μm pitch whilst 100 μm islands were organised in an orthogonal lattice concentric to the 30 μm island lattice (Fig. 1b4) with a 200 μm pitch. Some of the islands in Fig. 1b4 are intentionally displaced to illustrate how easily they can be detached for the formation of functional bio-hybrids (see below). The charge injection limit of the active CNT electrodes was ∼2 mC/cm^2^ [34]. With large 3D surface areas, CNT islands are more efficiently coupled to biological tissue when hydrophilic. This was achieved through ionisation with Nitrogen plasma for 10 min followed by submersion in de-ionised H_2_O for at least 24 h prior to retinal interfacing.

### 2.2 Retinal isolation

Experimental procedures were approved by the UK Home Office, Animals (Scientific procedures) Act 1986. Cone rod homeobox (Crx) knockout animals were sacrificed by cervical dislocation followed by immediate enucleation. Isolation of the retina was performed in carboxygenated (95% CO_2_/5%CO_2_) artificial cerebro-spinal fluid (aCSF) containing (in mM): 118 NaCl, 25NaHCO_3_, 1NaH_2_PO_4_, 3KCl, 1MgCl_2_, 2CaCl_2_, 10C_6_H_12_O_6_, 0.5 l-Glutamine (Sigma Aldrich, UK). First, the cornea was pierced with a hypodermic needle (23G gauge, BD Microlance 3, USA). Then, a pair of Vannas scissors (WPI, USA) was used to shear through the cornea, along the ora serrata before removing the lens. The sclera was then peeled off, using two pairs of sharp forceps, exposing the retina. The vitreous humour was teased off using those forceps, taking great care not to pierce the retina or remove the ILM.

### 2.3 Electrophysiological setup

Isolated retinas were mounted onto MEAs with the RGC layer facing down onto the electrodes and the optic disc outside the MEA active area. To improve coupling between the tissue and the electrodes, a polyester membrane filter (5 μm pores) held the retina in place whilst being weighed down by two stainless steel anchors bearing a framework of parallel glass capillaries. Thirteen retinas were mounted onto CNT MEAs and six onto TiN MEAs (MultiChannel Systems, Reutlingen, Germany).

To preserve physiological conditions, the tissue was perfused with oxygenated aCSF at 1 ml/min over the course of up to 72 h using a peristaltic pump (SCI400, Watson Marlow, UK). During the recording of electrophysiological activity, retinal explants were maintained at 32 °C using a temperature controller (TC02, MultiChannel Systems, Reutlingen, Germany) regulating a metallic plate below the MEA (MEA1060 INV, MultiChannel Systems, Reutlingen, Germany) and an inline heater for the inflow of aCSF (Ph01, MultiChannel Systems, Reutlingen, Germany). To prolong the viability of the tissue, retinas were kept at room temperature overnight [35], [36].

Although Crx−/− mice do not develop photoreceptor-mediated vision, melanopsin-induced phototransduction occurs in intrinsically photosensitive RGCs in the presence of blue light [37]. To avoid activation of these cells, all experiments were carried out in complete darkness.

Electrophysiological signals were recorded using 60-channel MEAs interfaced with a computer running the proprietary software MC_Rack (MultiChannel Systems, Reutlingen, Germany) using an MEA1060INV (Multi Channel Systems, Reutlingen, Germany) amplifier via a PCI MCS card.

Recordings were acquired at 25 kHz sampling frequency. Spontaneous activity was recorded for 10 min before and after each stimulation run, amounting to 15 stimulation runs and 15 spontaneous activity files per retina. Electrical stimulation of retinal tissue was achieved via individual electrodes on the MEA connected to an STG2002 stimulator (Multi Channel Systems, Reutlingen, Germany) controlled by a computer running the proprietary software MC_Stim (Multi Channel Systems, Reutlingen, Germany). The stimulator was also connected to the recording equipment PCI acquisition card for synchronised recordings. Acquisition of electrophysiological signals during stimulation runs started 100 ms before the stimulus and ended 200 ms after the stimulus.

Retinas were electrically stimulated by rectangular charge-balanced biphasic pulses with a 30 μs inter-phase interval (Fig. S2). The charge delivered by each pulse was modulated by altering either the current intensity (10–100 μA per phase) or phase duration (10–100 μs per phase).

### 2.4 Analysis of electrophysiological data

MCD files were imported into Matlab (The MathWorks, USA) using the FIND toolbox [38]. The artefact introduced by applying electrical stimulation often saturates the amplifier for a few ms, followed by a large voltage deflection. This artefact was removed by local curve fitting using the SALPA algorithm [39]. Spikes were automatically extracted, then sorted by supra-paramagnetic clustering and wavelet analysis using Wave_clus [40].

Epochs containing sorted spikes for each electrode were mapped to the different stimulus conditions and organised as raster plots with the ordinates divided into the different charge injected values in ascending order. Consequently, the results for a single stimulation run consisted of a set of six (the number of different stimulating combinations) raster plots for each spike cluster on each recording electrode.

**Fig. 2:**
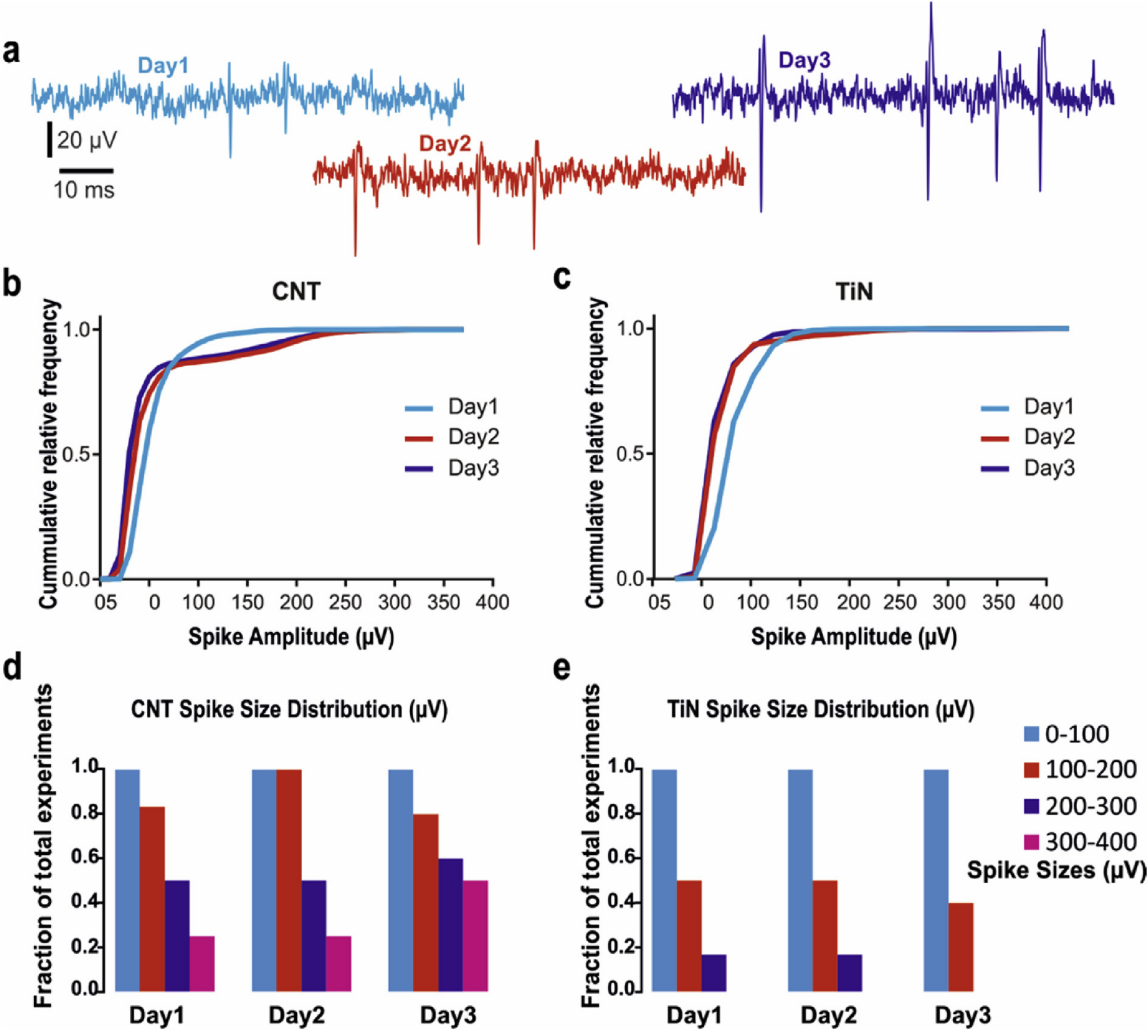
**Acute increase in spike amplitude**. **(a)** Example of raw signals recorded on one electrode over three consecutive days, displaying an increase in spike amplitudes and signal to noise ratio over time. **(b**–**c)** Relative cumulative distribution of spike amplitudes for a single retina on a CNT **(b)** and TiN **(c)** MEA. **(d**–**e)** Fraction of retinas with RGCs exhibiting spike sizes of varying amplitudes (0–400 μV), arranged in 100 μV bins. Spikes with amplitudes up to 400 μV were observed on all three days for CNT electrodes **(d)** but not for TiN electrodes **(e)**.

Evoked responses were identified on the raster plots by their synchronicity in repetitive trials of a given condition and by their disappearance below a given amount of injected charge), determining the firing threshold for eliciting spikes (Fig. 2d). Spike sizes were measured using the peak-to-peak amplitude of each extracted waveform. Statistical analyses and graph plotting were carried out using Sigma Plot (Systat Software Inc., San Jose, California, USA) and Excel (Microsoft, USA) respectively. Statistical significance was measured using non-parametric analyses with a significance limit of p ≤ 0.05.

### 2.5 Viable retina-CNT biohybrids

In order to investigate the morphological coupling between CNT constructs and the inner retina, retinas were incubated laid on passive CNT discs loosely attached to their Si/SiO_2_ substrate (Fig. S4). Incubation chambers consisted of a 100 mm diameter Petri dish with multiple (4 and above) plastic rings (6 mm height, 10 mm diameter) affixed to the bottom with polydimethylsiloxane (PDMS), a biocompatible silicone adhesive [41]. Passive CNT devices were glued to the bottom of Petri dishes. In order to render the CNT islands hydrophilic, incubation chambers were initially ionised with Nitrogen plasma for 10 min and submerged in de-ionised H_2_O for 24 h prior to retinal incubation in aCSF. Isolated retinas were placed, RGC layer down, onto CNT assemblies. As for MEA preparations, retinas were held in place with a membrane filter and stainless steel anchor weights. aCSF was perfused into the main chamber (0.5 ml/min) and carboxygenated directly within the chamber. To test the viability of the tissue, some biohybrids were removed after 72 h and placed onto an active MEA and they always displayed both spiking activity and local field potentials on multiple channels.

### 2.6 Electron microscopy

For TEM, retinas incubated onto loose CNT discs were lifted off from the SiO_2_ substrate using a piece of nitrocellulose membrane filter, halved along the optic disc, then fixed in 2% Glutaraldehyde (Cacodylate buffered), post-fixed in osmium tetroxide, dehydrated in acetone, and embedded in epoxy resin (TAAB, UK). Semi-thin (2 μm) and ultra-thin (70–90 nm) sections were cut perpendicular to the surface of the retina on an Ultracut E ultra-microtome (Reichert-Jung, Austria, now Leica Microsystems), then stained with either Toluidine Blue (semi-thin sections) or uranyl acetate and lead citrate (ultra-thin sections). Semi-thin sections were obtained in levels of 3 series comprising 6 sections to be used for staining to locate the CNT islands in the tissue. Thus, each one of these levels corresponded to a 36 μm-long segment of tissue, preventing the inadvertent full sectioning of a large CNT island.

For scanning EM, retinas incubated onto loose CNT discs were lifted off from the SiO_2_ substrate using a piece of nitrocellulose membrane filter, halved along the optic disc, then fixed in 2% Glutaraldehyde (Sorensons buffered), dehydrated in increasing concentrations of ethanol and critical-point dried (CPD 030, Bal-Tec, Lichtenstein, now Leica Microsystems). The samples were coated with a 15 nm gold film using a Polaron E5550 sputter coater (Polaron equipment Ltd, UK, now Quorum Technologies Ltd) before observation in a Cambridge Stereoscan 240.

### 2.7 Immunohistochemical labelling of retinal sections

Mouse eyes were enucleated, fixed for an hour in 4% paraformaldehyde, cryo-protected in a 30% sucrose phosphate buffered saline (PBS) solution for 24 h, embedded in Tissue-Tek OCT Compound (Sakura, Japan) at −30 °C and sectioned in 10 μm slices with a cryostat (HM560, Microm International). Sections were gathered on gelatine-coated slides, air-dried for 24 h, immersed in de-ionised H_2_O for 1 min to dissolve the OCT compound and air-dried for 1 h before immunohistochemical labelling.

Slides were kept at room temperature (if not specified otherwise) in a humidifying chamber and washed 3 times for 5 min in 0.1 M PBS (phosphate buffered saline) between each of the following steps in which reagents were diluted in a solution consisting of 0.1% Triton X 100 (Sigma-Aldrich, UK) and 0.1 M PBS: sections were exposed to 0.6% H_2_O_2_ for 20 min (to remove endogenous peroxidases), 20% blocking serum (Normal Horse Serum, Vector Labs, UK) for an hour, 2.5 μl/ml mouse anti-GFAP and 10% blocking serum for 16 h at 4 °C, 2 μl/ml biotinylated anti-mouse IgG (Vector Labs, UK) for 3 h, 2 μl/ml horseradish peroxidase-streptavidin (Vector Labs, UK) for 3 h then finally reacted with H_2_O_2_ and DAB.

Nuclear staining was achieved by dipping slides in a solution of Mayer's Haematoxylin before dehydrating the tissue in increasing concentrations of ethanol (50%, 70%, 90% then twice 100% for 3 min each), 3 min in 2 consecutive jars of Histoclear (Fisher Scientific, UK) and finally, coverslipped using Histomount (Fisher Scientific, UK).

### 2.8 Immunohistochemical labelling of retinal wholemounts

Retinas were fixed in 4% PFA for 1 h then washed 3 times for 5 min in de-ionised water. These were then placed, RGC layer facing up, on gelatine-coated slides and dried overnight. Slides were kept at room temperature (if not specified otherwise) in a humidifying chamber and washed 3 times for 5 min in 0.1 M PBS between each of the following steps in which reagents were diluted in a solution consisting of 0.1% Triton X 100 (Sigma-Aldrich, UK) and 0.1 M PBS: sections were exposed to 20% blocking serum (normal goat serum and normal donkey serum, Sigma Aldrich, UK) for 1 h, 5 μl/ml primary antibodies (mouse anti-GFAP, Sigma Aldrich; rabbit anti-laminin, ABcam) and 10% blocking serum for 20 h at 4 °C, 2 μl/ml secondary antibodies (FITC donkey anti-mouse and Cy3 goat anti-rabbit, Stratech, UK) for 3 h. After another PBS wash, slides were coverslipped with hard setting Vectashield (Vector labs, UK), then dried overnight.

### 2.9 Imaging of immunohistochemically stained retinas

Wholemount retinas were visualised with a Nikon A1R point scanning confocal microscope (Nikon Corporation, Japan) with the RGC layer facing the objective. Tiff stacks were processed in ImageJ (NIH, USA). Peroxidase stained retinal sections were visualised on an Olympus BX60 (Olympus, Japan) microscope with a Zeiss Axiocam HRc camera using Zeiss Axio Vision 3.1 software (Carl Zeiss AG, Germany). Fluorescence-labelled retinal sections were acquired on a Leica DMRA microscope (Leica Microsystems) with a Hamamatsu Orca-ER camera (Hamamatsu Photonics, Japan) using the Axio Vision 4.8 (Carl Zeiss AG, Germany) software. The average number of CNT islands promoting reactive gliosis per explant was calculated by counting the number of islands (within each size category) in each explanted retina which had above-background fluorescence within 20% of its circumference and dividing it by the total number of islands observable within that explant. This value was then averaged across all explants.

## 3 Results

### 3.1 Electrophysiological evidence for acute time-dependent increase in coupling of CNT electrodes

To evaluate the performance of CNT electrodes, we compared the electrical behaviour of RGCs coupled to CNT electrodes versus TiN electrodes. CNT electrodes yielded larger signals than TiN electrodes, displaying significantly larger spike amplitudes after a few hours *in vitro* (Fig. 1c). RGCs were stimulated above firing threshold using charged-balanced biphasic current pulses (see Methods). It was not possible to record from the stimulating electrode due to amplifier saturation within the first few milliseconds post-stimulation (Fig. 1g). To differentiate between responses unequivocally elicited by direct stimulation of the spiking RGC from those elicited through stimulation of secondary retinal interneurons making functional connections with the RGCs under investigation, responding RGCs were pooled according to response latency. Responses occurring within 10 ms of electrical stimulation were classified as direct and those occurring after more than 10 ms were classified as indirect (Fig. 1d). The fraction of direct responses was significantly higher than that of indirect responses for both CNT and TiN electrodes (Fig. 1e) pooled across all recording time points per explant. For the rest of this paper, we concentrate only on these more frequent direct responses.

### 3.2 Age-dependent increase in RGC stimulation thresholds in dystrophic retinas

Retinal degeneration can occur naturally (e.g. Royal College of Surgeons rat [42]), be due to genetic alterations or initiated by a physical insult (e.g. retinal detachment or exposure to strong light in albinos). Rodent models of outer retinal dystrophies are widely used [43], [44] to reach a better understanding of the kinetics of retinal degeneration and designing appropriate biological rescue strategies.

Although the kinetics of degeneration differ for each type of retinal dystrophy, retinal outer degenerative disorders follow the same general pattern [44]. In this study, we have used the Crx knockout mouse, a model of Leber congenital amaurosis. Crx is an important gene for the survival of photoreceptors and for the development of their outer segments [31], [45]. Photoreceptors in the Crx−/− mouse are born with atrophied outer segments and they cannot transduce light. They begin to die at postnatal day (P) 30 and the outer nuclear layer progressively shrinks from 12 to 1 layer by P210 [45], resulting in much thinner retinas than normal (Fig. S1). Substantial inner retinal remodelling occurs during that period. The glial scarring associated with degeneration of the Crx−/− retina (Fig. 1f) can push stimulating electrodes away from their target RGCs, thus increasing the charge required to bring these neurons to firing threshold.

In order to assess the impact of age-related retinal gliosis on epi-retinal stimulation threshold values, we stimulated RGCs from the Crx−/− retina between birth and 4 months of age. Fig. 1h displays the longitudinal changes in average (±SEM) charge thresholds for both TiN and CNT electrodes on progressively ageing (and thus degenerating) retinas of the Crx −/− mouse. This shows that there is a pronounced increase in threshold charge as degeneration progresses and the neural retina becomes increasingly entangled in glial processes (Fig. 1f). However, the increase in threshold is much less pronounced when using CNT electrodes than when using standard TiN electrodes, with the difference becoming statistically significant at advanced degeneration stages. These results already demonstrate that CNT electrodes offer a more efficient interface for cellular stimulation, presumably because they allow the formation of more intimate contacts between the tissue and the prosthetic device (see below).

### 3.3 Spike amplitudes increase with time

The size of action potentials is an indication of the quality of the coupling between the retinal tissue and the recording electrode, as the propagation of such signals through resistive biological tissue is attenuated with increasing distance between the two. We have previously reported that spike waveforms increase in size within the first hour after mounting the retina on a CNT MEA [16]. In this study, we have followed these changes for up to three days *in vitro*, to detect mechanical, electrophysiological and histological changes.

Fig. 2a shows two examples of signal improvement over 8 h on two channels from a CNT array, clearly illustrating that the waveforms become larger with time upon contact with CNT electrodes. Fig. 2b, c illustrates the cumulative distribution of spontaneous spike amplitudes recorded on a CNT (Fig. 2b) and TiN (Fig. 2c) MEA over three days for one retina (see also Fig. S3 for non-normalized, non-cumulative distributions of spike amplitudes over three days). There is a clear shift towards larger amplitudes on Day 2 and Day 3 for the spikes recorded on the CNT MEA, but not for spikes recorded on the TiN MEA. Fig. 2d, e summarizes changes in spike sizes over time for all retinas, clearly showing that CNT, but not TiN electrodes yielded larger signals on Day2 and Day3 than on Day1. Moreover, spikes with amplitudes as large as 400 μV could be seen with CNT electrodes, whereas signals recorded with TiN electrodes never reached such large amplitudes.

### 3.4 Stimulation thresholds decrease with time

17 retinas from animals aged between P80-110 were experimented on over a period of three days (6 on TiN and 11 on CNT MEAs), stimulating the same four individual electrodes (for each retina) on each day, generating a theoretical total of 204 stimulation runs. However, some experiments did not successfully yield data for three consecutive days (e.g. artefacts too strong to be efficiently removed, tissue deterioration on Day 3, human error during experimental procedure), leading to a total of 168 stimulation runs (109 for CNT and 59 for TiN electrode experiments).

Stimuli with a cathodic initial phase and an asymmetric shape consisting of a reversal phase twice as long and with half the current amplitude of the initial phase were significantly more efficient at activating RGCs (lower threshold charge, Fig. S2) than other stimulus shapes both for CNT and TiN electrodes.

**Fig. 3:**
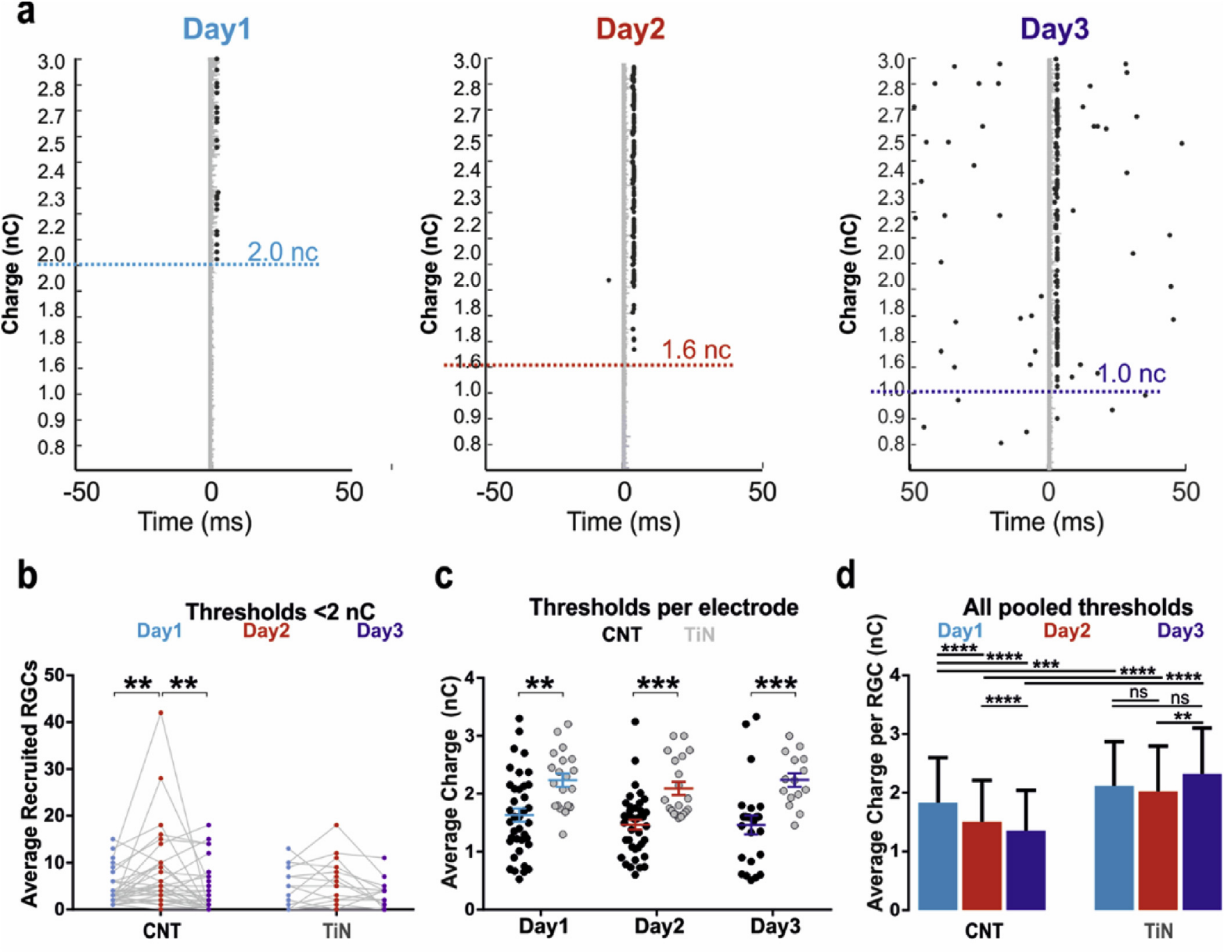
**Changes in stimulation thresholds, spike amplitudes and cellular recruitment over the course of 3 days *in vitro***. **(a)** Raster plots of direct electrical stimulation of an RGC on a CNT MEA, displaying a gradual decrease in charge threshold over time. Epochs are organised by increasing amount of stimulation charge value along the ordinate axes. These are not linear or unique as they represent the product of multiple parameters for stimulus current and single-phase duration (see methods). Grey traces at time zero (stimulation onset) indicate for each trial periods during which the signal was irretrievably corrupted by stimulation artefact. **(b)** Average number of recruited RGCs with thresholds below 2 nC per electrode on Days 1–3 for both CNT and TiN MEAs. Asterisks indicate statistical significance (Bonferroni's multiple comparisons test), with p = 0.0081 and p = 0.0022 for CNT Day1-Day2 and Day2-Day3, respectively. N = 34. **(c)** Average threshold values per electrode recorded on Days1–3 for both types of MEAs. Asterisks indicate statistical significance (Sidak's multiple comparisons test), with p = 0.0013, p = 0.0008 and p = 0.0006 for Day1 Day2 and Day3, respectively. For CNT electrodes, N = 38, N = 39 and N = 23 for Day1, Day2 and Day3, respectively. For TiN electrodes, N = 20, N = 20 and N = 15 for Day1, Day2 and Day3, respectively. **(d)** Average (±SD) threshold (nC) across all RGCs recorded in this study. Asterisks indicate statistical significance (Dunn's multiple comparisons test), with ***p < 0.0001 and **p = 0.0059. N = 241 (CNT Day1), N = 389 (CNT Day2), N = 155 (CNT Day3), N = 230 (TiN Day1), N = 287 (TiN Day 2) and N = 148 (TiN Day3).

Fig. 3a illustrates an example of direct responses in a single RGC, requiring an increasingly lower amount of charge for activation over time, whilst concurrently spikes increase in amplitude (Fig. 2a). The fraction of responding RGCs with thresholds < 2 nC out of all spiking RGCs per retina increased significantly on Day2 for CNT electrodes but not for TiN electrodes (Fig. 3b). The average threshold value yielded by CNT electrodes was also significantly lower on each day compared to values obtained with TiN electrodes (Fig. 3c). Finally, when stimulating with CNT electrodes, the average direct threshold values became gradually and significantly lower from Day1 to Day3 (Fig. 3d) with no significant changes over time for TiN electrodes. For both electrode materials, fewer cells participated in the responses on Day3, presumably because of deteriorating health of the tissue. These results indicate a physical decrease in the resistivity between CNT electrodes and RGCs, suggesting that the retina becomes increasingly more intimately coupled to the electrodes.

### 3.5 Ultrastructural evidence for time-dependent increase in coupling of CNT electrodes

**Fig. 4:**
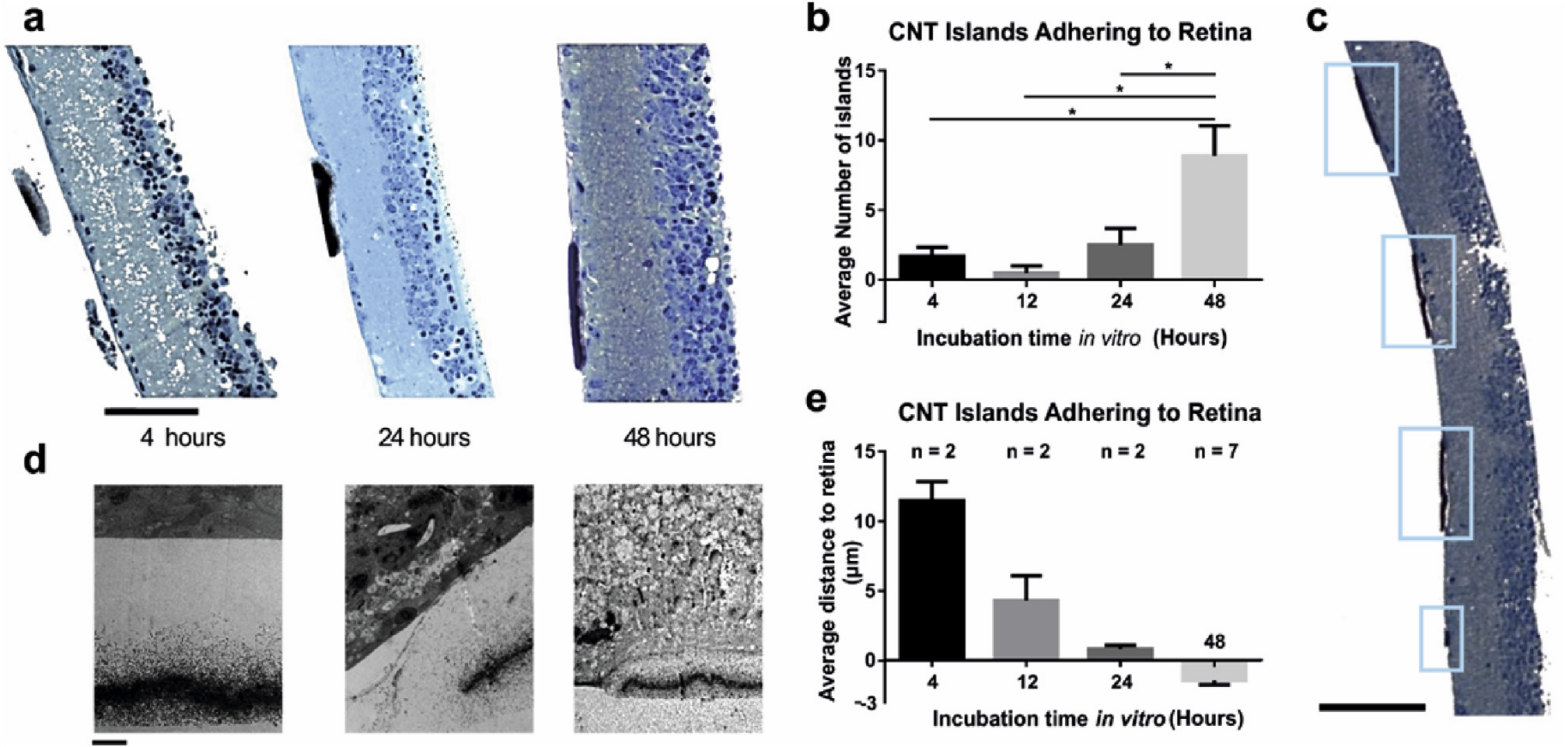
**Morphological evidence of CNT electrodes integration into dystrophic retinas over the course of 3 days**. **(a)** Semi-thin transverse sections stained with Toluidine blue demonstrate the progressive integration of CNT islands into the inner retina over three time points (4, 24 and 48 h). Scale bar is 100 μm. **(b)** Bar graphs demonstrating the progressively higher number of large CNT islands adhering to retinas with time (average ± SEM). Asterisks indicate statistical significance (Mann-Whitney test), with p = 0.0318, p = 0.0364, p = 0.0434 for 48 h vs 4, 12 and 24 h, respectively. **(c)** Example semi-thin section with four CNT islands adhering to its surface (blue boxes). Scale bar is 100 μm. **(d)** Transmission Electron Microscopy (TEM) micrographs showing the ultrastructural details of the integration described in **(a)**. Scale bars are 2.54 μm (4 h), 3.36 μm (24 h) and 8.4 μm (48 h). **(e)** Bar graphs demonstrating the progressively shorter distance between large CNT islands and retinal tissue with time (average ± SEM). The negative values highlight the fact that portions of the islands are physically engulfed in the ILM. It was not possible to perform statistical tests at short incubation times because too few CNT islands adhered to the retina. (For interpretation of the references to colour in this figure legend, the reader is referred to the web version of this article).

The morphological and anatomical effect of CNT assemblies on the inner retina were investigated with immunohistochemical (IHC) labelling and electron microscopy performed on retinal explants which had been interfaced with CNT constructs for 4, 12, 24 and 72 h (see Methods and Fig. 4 and Fig. S4). CNT islands initially loosely attached to their Si/SiO_2_ substrate were stripped off as they became gradually embedded in the retina. Semi-thin sections of retinal bio-hybrids were used to quantify the physical coupling between the tissue and CNT islands through the average number of islands adhering to retinal explant (Fig. 4b) and the distance between the external surface of each island and the retinal inner limiting membrane (ILM) (Fig. 4e). 100 μm CNT islands gradually integrated into the ILM over 48 h (Fig. 4a–e), evidenced by a decrease in the distance between the CNT islands and the tissue (Fig. 4a, d, e) and by an increase in the number of islands adhering to the retina (Fig. 4b). As there were only few 100 μm adhering islands at shorter time points, we could not perform reliable statistical analysis on that particular dataset. Fig. 4c illustrates multiple adhering CNT islands following 48 h retinal interfacing.

TEM performed at each of these four time points revealed a more detailed picture of the integration of CNT islands within the ILM. After 4 h, there was no direct contact between individual CNTs and the ILM (Fig. 4d, left panel). Indeed, the closest CNT we found to the ILM was approximately 2 μm away (Fig. S5). We also observed some strands of biological material (Fig. S5) between the CNTs and ILM, suggesting that some remnants of the vitreous humour (probably collagen fibrils) were buffering the zone between the electrode and the retina. After 12 h, we found evidence of thickening of the vitreous between the retina and the CNT island. Fig. S6 illustrates this process, with portions of the vitreous closer to the retina appearing denser than those further away. Indeed, there are fewer and smaller gaps (white portions of the micrograph which have not been stained with osmium tetroxide, Uranyl acetate or lead citrate) in the vitreal matrix in panel f than in panel e of Fig. S6. After 24 h in contact with CNT islands, the retina changed shape to accommodate the island (Fig. S7). We also observed evidence for the formation of an accessory limiting membrane (Fig. 4d and Fig. S7) which grasped the CNT island from the edges and on occasions, we could see individual CNTs penetrating the ILM. This was observed on multiple sections on two different explants.

**Fig. 5:**
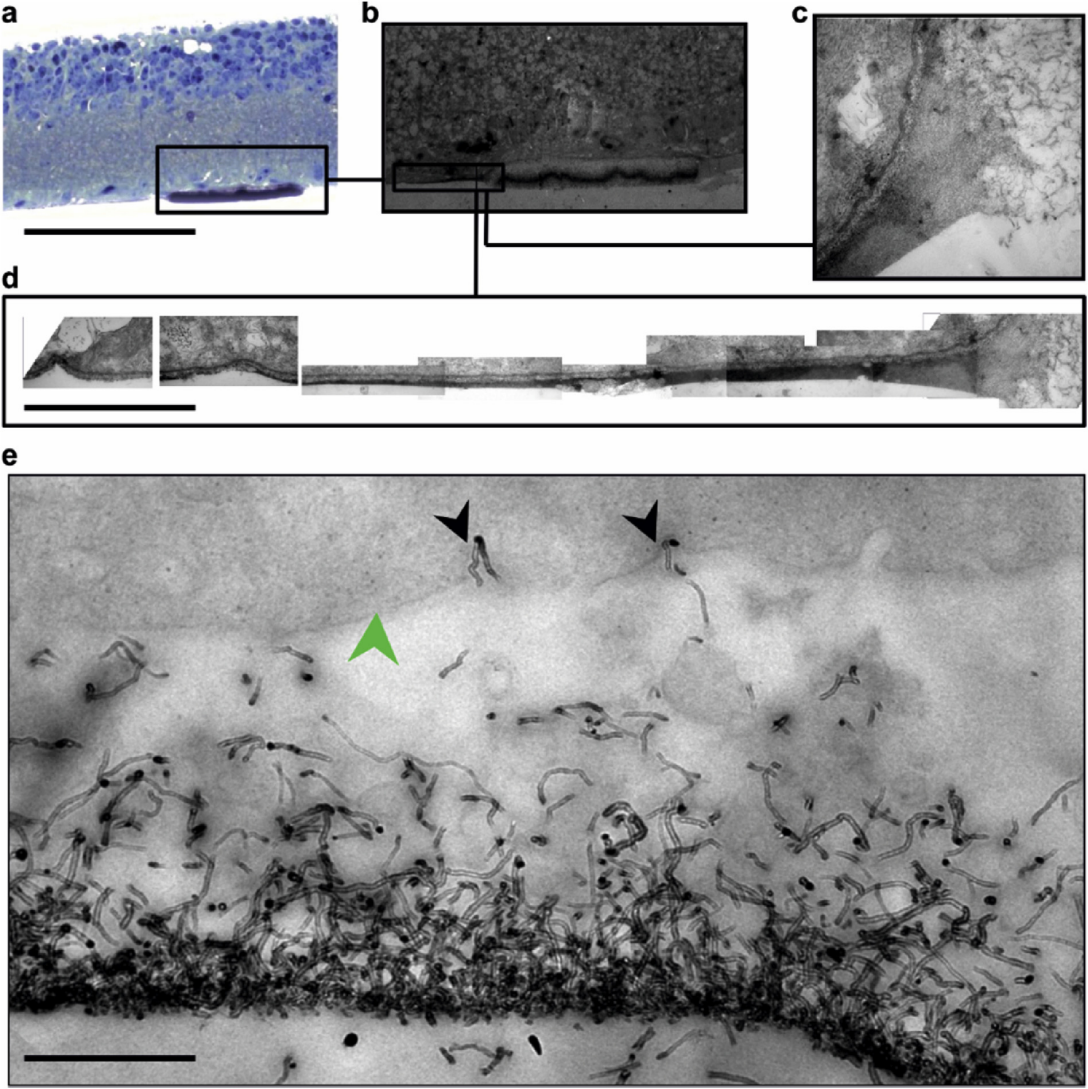
**Homogenous matrix grappling edge of CNT island after 48 h *in vitro* extends along the retina for over 24 μm**. **(a)** semi-thin transverse section stained with Toluidine blue. **(b)** TEM micrograph of the same retina with the CNT island grasped by a long homogenous matrix (box). **(c)** Higher magnification micrograph showing the homogenous matrix grabbing the edge of the CNT island. **(d)** digitally-stitched collage of 10 TEM micrographs which follow the matrix to an invagination in the ILM 23 μm away. **(e)** Individual CNTs (black arrowheads) penetrating the retinal ILM (green arrowhead). Scale bar is 50 μm in **(a)**, 60 μm in **(b)**, 3 μm in **(c)**, 4.5 μm in **(d)** and 500 nm in **(e)**. (For interpretation of the references to colour in this figure legend, the reader is referred to the web version of this article).

After 48 h, islands were completely integrated into the ILM (Fig. 4d), with collagen fibrils from the lamina rara externa anchoring individual CNTs (Fig. S8) and the accessory limiting membrane described above extending for tens of microns. Fig. 5 shows a transverse profile of how the retinal tissue interacts with a CNT island at increasingly higher magnification. Panel (a) shows a light micrograph of a Toluidine Blue semi-thin section, already clearly indicating that the CNT island has become integrated in the retinal tissue, which is more conspicuous on the lower power TEM in panel (b). Panel c is a magnification of the edge of the CNT island, where individual CNTs can be seen enveloped in a homogenous matrix. This matrix can be seen extending for tens of microns along the ILM (d), becoming progressively thinner as the distance from the CNT island edge increases. We also observed multiple instances of CNTs penetrating the retinal tissue (Fig. 5e).

### 3.6 Morphological adhesion of CNT constructs onto retinal wholemounts

**Fig. 6:**
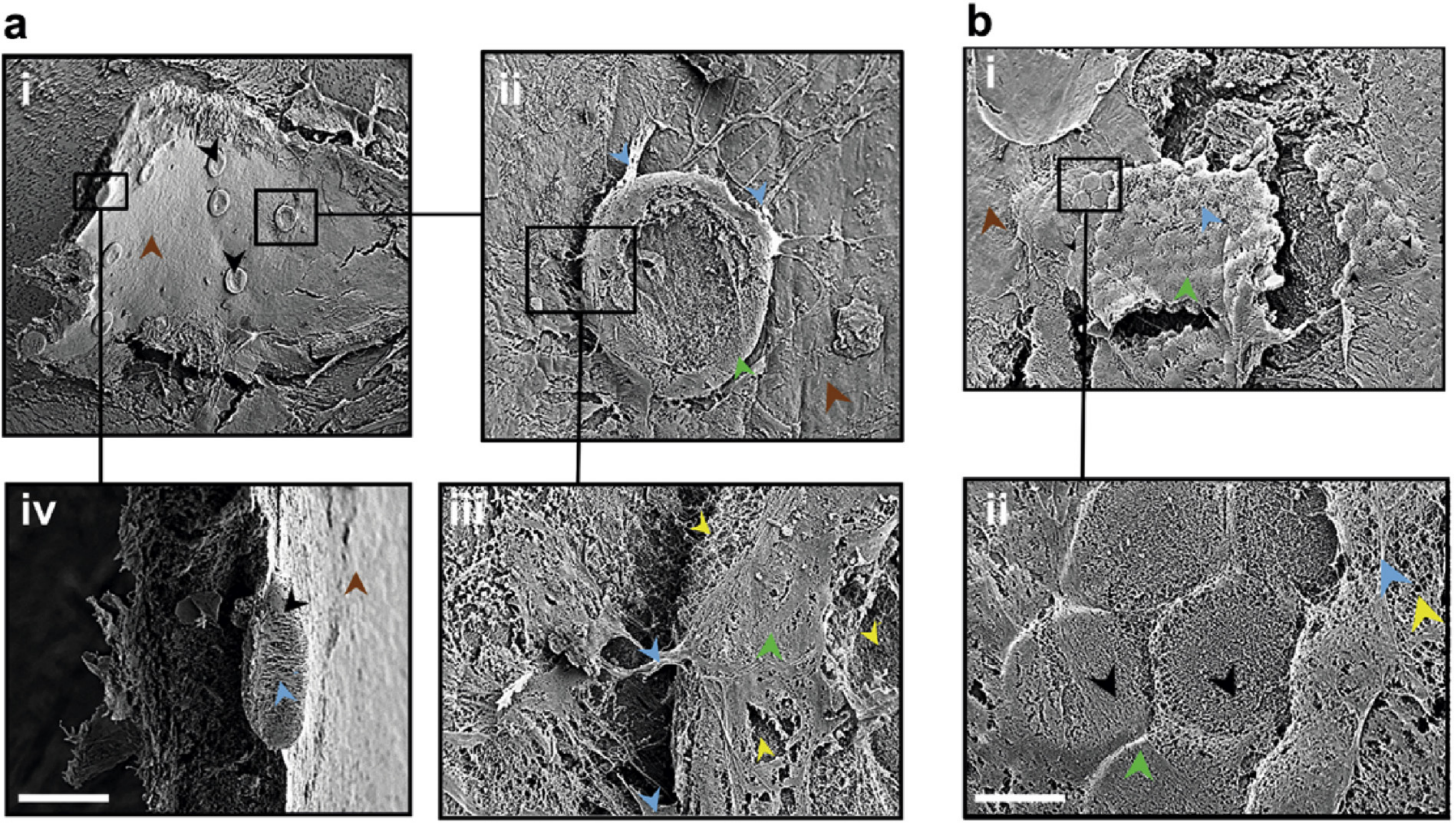
**SEM images of the ILM incorporating large CNT islands**. **(a)** Retinal explant with 10 large CNT islands (black arrowheads) adhering to its ILM (brown arrowheads). Scale bars are 847.6 μm (top-left quadrant), 100 μm (top-right quadrant), 125.35 μm (bottom-left quadrant), and 23.42 μm (bottom-right quadrant). **(b)** Retinal explant with over 50 small CNT islands (black arrowheads) embedded into an accessory limiting membrane (green arrowhead) continuous with the ILM (brown arrowhead). Scale bars are 200 μm (top panel) and 27.38 μm (bottom panel). (For interpretation of the references to colour in this figure legend, the reader is referred to the web version of this article).

**Fig. 7:**
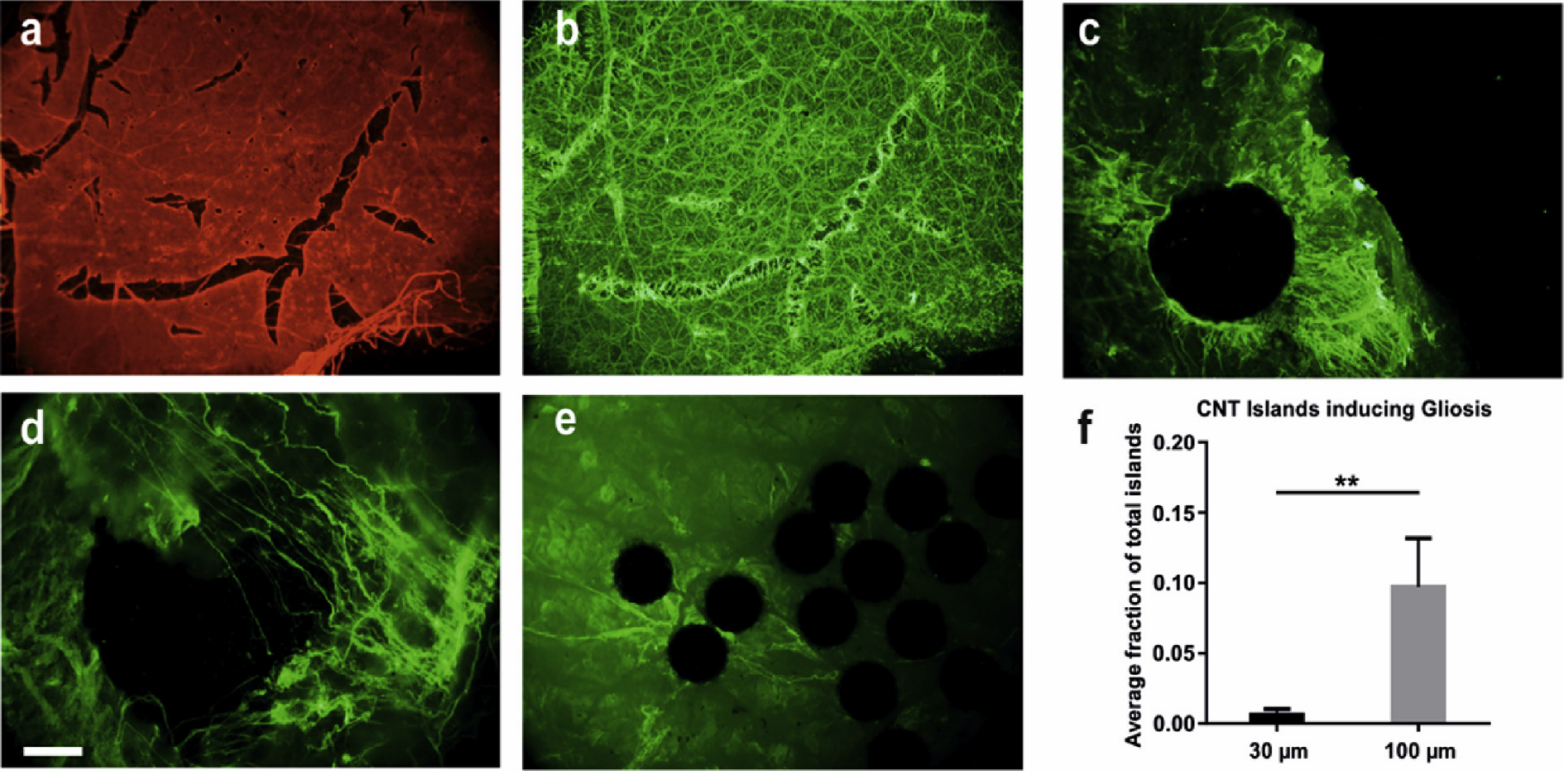
**Immunohistochemical analyses of CNT-retinal interactions**. **(a**–**b)** Micrographs of control retinal wholemounts stained with laminin **(a)** and GFAP **(b). (c**–**d)** Higher magnification micrographs of retinal wholemounts interfaced with 100 μm CNT islands and stained for GFAP, displaying activation of both astrocytes **(c)** and Müller cells **(d)**. **e)** micrographs of retinal wholemounts interfaced with 30 μm CNT islands and stained for GFAP. **(f)** Bar graph displaying the average fraction of islands inducing reactive gliosis. Asterisks indicate significance (Mann-Whitney test), with p = 0.0064, N = 7 explants. Scale bar is 75 μm for **(a**–**b)**, 30 μm for **(c)**, 16 μm for **(d)** and 12 μm for **(e)**.

Scanning electron micrographs (SEM) of explants incubated over 48 h revealed both 100 μm (Fig. 6a) and 30 μm (Fig. 6b) CNT islands incorporated into the ILM. Panel (ii) of Fig. 6a displays an explant with ten 100 μm islands adhering to its surface, covered by the ILM which has a characteristically smooth appearance in SEM (Fig. S1). The two middle panels focus on one of the islands at higher magnification, revealing fibrous bundles (blue arrows) clutching the side of the island. These fibre bundles can reach tens of microns in length, perhaps reflecting the long matrix described in Fig. 5, although they may also reflect activated glial cells (see Fig. 7c, d). Panel (iii) focuses on part of the island periphery, which appears to be covered in an accessory limiting membrane with a texture somehow similar to the ILM (green arrowhead), with gaps exposing the CNTs underneath (yellow arrowheads). Panel (iv) focuses on a CNT island which has been fractured following critical point drying (Methods), allowing us to visualize biological bundles (as described above) infiltrating the CNT island throughout.

Fig. 6b displays a portion of the retina covered by more than fifty 30 μm CNT islands, forming a hexagonal lattice incorporated into the retina. Some islands are not completely covered (ii), allowing visualization of individual CNTs (yellow arrowhead) below ILM fibres (blue arrowheads) in the process of weaving the accessory limiting membrane.

### 3.7 Immune reaction of retinal tissue to interfacing with CNT constructs

Immunohistochemical labelling of wholemount retina-CNT biohybrids with Glial Fibrillary Acidic Protein (GFAP) allowed to estimate the impact CNT assemblies have on the biochemical integrity of inner-retinal tissue glia. In response to an invasive threat, glial cells are known to activate, a process characterized by the upregulation of GFAP [21], [46] and followed by various cellular responses which can be detrimental to, and even reject an implanted device [47]. In the dystrophic retina, Müller glia are in a state of activation and heavily involved in the remodelling process associated with degeneration [48], [49]. Tears in the ILM lead to reactive gliosis, emphasizing the requirement for gentle interfacing between epi-retinal electrodes and ILM. Fig. 7a displays a positive control for gliosis, with fluorescent double labelling for laminin (one of the main components of the ILM) and GFAP, highlighting an increase in GFAP levels coinciding with the areas showing tears in the laminin layer. The average number of 100 μm CNT islands initialising reactive gliosis per retina was significantly larger than for 30 μm islands (Fig. 7f). 100 μm CNT islands induced both astrocytic (Fig. 7c) and Müller gliosis (Fig. 7d) whilst 30 μm islands were rarely seen to promote a glial response (Fig. 7e).

## 4 Discussion

The ability to safely stimulate RGCs requires implanted electrodes to deliver supra-threshold charge over prolonged durations without causing damage to the tissue or electrode material. Although this goal has been generally achieved for retinal prosthetics, one of the technological hurdles remaining is the size of the electrodes, with smaller electrodes leading to higher spatial resolution. But electrodes with a smaller surface also require higher stimulation charge density to reach supra-threshold levels. Not only are electrode materials intrinsically limited by the density of charge they can store at their surface, but high charge densities can lead to electrode and tissue damage [4], [48], [49]. Lower charge injection during electrical stimulation would lead to more energy efficient prostheses, thus increasing the potential number of powered channels as well as battery life and computational power. Lower charge densities can be achieved by (1) using “optimal” stimulating electrode materials with a larger surface area; (2) using optimal stimulation protocols requiring less charge to depolarise target cells; and (3) reducing the distance between electrode and target neurons. These points will be discussed below with reference to the data presented in various studies, including the present one (summarized in Table 1).

### 4.1 Optimal stimulating electrode materials

The reversible charge storage capacity (CSC) is the amount of charge per unit geometric surface area that an electrode can store at its surface, and thus deliver in the leading phase of a stimulation pulse. It depends on the electrode material, its size and shape, composition of the electrolyte, and the parameters of the stimulation waveform. As such, materials with large effective surface areas make optimal stimulating electrodes.

Some materials operate through faradaic reactions to deliver charge to the tissue. Noble metals such as platinum or platinum iridium alloys confine the faradaic reactions to a monolayer at the surface of the electrode, giving them the name of pseudo-capacitive electrodes [50]. For example, TiN exhibits a rough columnar topography and has a charge injection limit estimated at 3.7 mC/cm^2^ [51]. A new generation of stimulation electrodes make use of three dimensional multivalent coatings capable of undergoing reversible redox reactions, yielding high charge injection capacities. Examples of such coatings are IrO_2_ and polyethylenedioxythiophene (PEDOT), with CSCs estimated at 28 mC/cm^2^ and 75 mC/cm^2^, respectively [52]. Nanocrystaline diamonds have recently been considered for retinal stimulation showing promising biocompatibility [53] and a theoretical CSC of 10 mC/cm^2^ when topographically enhanced with CNTs [54].

For stimulating electrodes, CNTs offer a number of substantial advantages over their metal-based counterparts. Mainly, their fractal-like geometry presents a large surface area, significantly increasing the charge injection capacity and lowering the electrode impedance value [55]. They also possess outstanding electrical [9], [10], chemical and mechanical properties as well as high tensile strengths [12], biocompatibility [13] and conductivity [14]. Conventional MEAs can be easily coated with solutions of single wall CNTs and used for the recording of action potentials in RGCs [56]. A recent study demonstrated the functionalization of PEDOT with single wall CNTs for retinal stimulation [51], achieving thresholds as low as 1 nC for PEDOT-CNT electrodes and 5 nC for TiN electrodes. The coatings described in those two studies are ideal for *in vitro* proof of concept experiments, but may delaminate over time, making them unsuitable for long-term clinical applications. The CNTs constituting our electrodes are grown by CVD directly into the porous TiN base, making them highly stable and unable to delaminate as a result of sustained electrical stimulation.

### 4.2 Optimal stimulating protocols

Epi-retinal stimulation of second order retinal neurons leading to synaptically driven responses has recently generated some interest [51], [58], [60], [64] with the optimisation of stimulation protocols based on longer pulses [65]. However, because of anatomical evidence pointing to remodelling of the inner retina during degeneration [43], [44], [59] combined with the fact that long stimulation pulses lead to irreversible faradaic reactions [66], we chose to restrict our stimulation parameters to shorter pulses (<100 μs), targeting RGCs directly. Although CNT electrodes proved effective over short stimulation timescales, stimulus pulses longer than 500 μs led to amplifier saturation, making it impossible to calculate the chronaxie and establish a rheobase.

Our stimuli were always charge balanced to avoid irreversible faradaic reactions at the electrode-electrolyte bilayer. However, the shape and polarity of each stimulus was delivered in a pseudo-random order. Our experiments yielded consistently lower thresholds when stimulating with (1) a cathodic first phase as well as (2) a slow reversal potential (second phase twice the duration and half the amplitude of the initial phase). A small proportion of our responses (<1%) consisted of axonal stimulation identified by their increase in voltage amplitude in response to increased charge as well as their synchronised appearance on multiple adjacent channels, typical characteristics of electrically evoked fibre bundle volleys.

Modelling studies demonstrate that RGC somata have lower thresholds than their axons [67] owing to the denser distribution of voltage gated sodium channels at the initial segment [68], [69]. This is supported by *in vitro* stimulation of the rabbit retina [57] with a 10 μm^2^ Pt-Ir extra-cellular electrode which yielded somatic RGC thresholds 50% lower than axonal thresholds. *In vitro* stimulation of rhesus monkey retinas with closely spaced Pt-plated electrodes (60 μm pitch, 9–15 μm diameter) presents evidence that the area most sensitive to epi-retinal stimulation is close to the soma and the proximal portion of the axon [63]. Patients implanted with the Argus I and II epi-retinal prostheses (Second Sight Medical Products, Inc, Sylmar, CA) report streak-like visual percepts rather than more commonly reported punctate-shaped phosphenes, suggesting direct stimulation of axonal bundles. This is to be expected considering that this study used very large electrodes (200 μm diameter) located far from the retina (∼180 μm), necessitating over 90 nC to reach RGC stimulation thresholds [7].

The use of much smaller CNT electrodes integrated to the patient's ILM would considerably reduce the amount of charge required to elicit phosphenes, and thus may contribute to reducing the probability of activating axonal responses. This was demonstrated by the same group *in vitro* with the use of smaller electrodes and longer stimulation pulses which were later used and validated in a patient [70].

### 4.3 Reducing RGC-Electrode distance

The present study has demonstrated the ability of CNT constructs to progressively integrate the inner retina whilst improving biophysical interactions between RGCs and electrodes without promoting a glial response. Indeed, gliosis was only experienced with 100 μm but not 30 μm CNT islands, which had a comparable size to the CNT electrode contacts in our electrophysiological experiments. The use of detachable CNT assemblies interfaced with the retina to estimate GFAP levels proved challenging. Indeed, as they are completely opaque, the CNT islands prevented the visualization of glial processes between CNTs and the RGC layer. However, we were able to visualize gliosis occurring at the edges of the CNT islands as well as “behind” them, i.e. on the vitreal surface of the retina, as glial cells appear to engulf the CNT islands (which is also suggested by our SEM pictures). Labelling retinal explants post recording was unfortunately not feasible because the process of removing the delicate retinal explants (these retinas are extremely thin, being devoid of photoreceptors) from the CNT contacts of the MEA resulted in severe lacerations in the retinal tissue, as CNTs were tightly bound to the ILM by the CNT “Velcro” effect demonstrated here through electron microscopy.

It is this Velcro effect, noticeable even just shortly after mounting the retina on CNT MEAs [16] that prompted us to investigate longer term effects. The ultrastructural results we present in this study demonstrate that 4 h after mounting the retina onto CNT arrays, there is no direct contact between CNTs and the ILM. This suggests that the most viscous portion of the vitreous is still firmly implanted into the ILM and may necessitate several hours of aCSF perfusion to dissolve away. One approach to improve implantation of a retinal prosthetic device would be to digest portions of the vitreous and ILM prior to implantation with glycosidic enzymes [71].

The strong adhesion that develops with time between the CNT assemblies and the vitreo-retinal interface can solve one of the fundamental problems with epi-retinal prosthetics, which is how to fix the prosthetic device to the retinal tissue. Currently, fixing arrays of stimulating electrodes to the inner retina requires the use of retinal tacks, which introduce a large gap between the electrodes and the tissue, resulting in very high stimulation (perceptual) thresholds, as high as 90 nC [7] (0.28 mC/cm^2^), a value several orders of magnitude higher than thresholds obtained in our study. Table 1 displays a list of studies investigating different types of electrodes for epi-retinal stimulation. The lowest values, with thresholds below 0.1 mC/cm^2^ are recorded in healthy retinas (except for Sekirnjac et al. [61], [62], [63], who were able to record evoked responses on the stimulating electrode as well by using a stimulation blanking system which we did not have for our study). This provides additional evidence as to the impact of reactive gliosis (discussed below) on epi-retinal stimulation of RGCs.

Most studies have shown that stimulation of dystrophic retinal tissue results in higher thresholds than in wild-type tissue (see Table 1). However, longitudinal studies of epi-retinal stimulation of age-matched wild type and P23H rat retinas aged between P37-752 did not reveal significant differences in response threshold or latency between both groups [61]. Further, no significant age-dependent changes were observed between the two groups.

As illustrated in Fig. 5 and Fig. S2, individual CNTs can penetrate the ILM, and possibly even individual RGCs. With appropriate nano-fabrication strategies and bio-functionalization of the CNT surface, this could be exploited further to achieve intracellular RGC stimulation in retinal prostheses, lowering the amount of charge necessary to elicit action potentials by several orders of magnitude. Residing directly between epi-retinal electrodes and their target cells, astrocytes represent a barrier to efficient direct RGC stimulation because a hypertrophic glial response to the insertion of a foreign body would widen the gap, thus increasing activation thresholds. Although quiescent astrocytes provide a neuroprotective environment for RGCs under normal conditions, they have been shown to promote RGC injury following activation [72]. A recent study has demonstrated that nanocrystalline diamonds with different terminations either enhanced (Hydrogen-terminated) or prohibited (Oxygen-terminated) protein coating, suggesting a design for micro-electrodes consisting of a protein coated base which would allow re-formation of the glial seal following penetration of the neural tissue by a non-coated penetrating tip with low impedance [73]. In the retina, the target neurons are protected by the ILM and surrounded by Müller cells, astrocytes and blood vessels. An optimal epi-retinal electrode would penetrate the ILM, bypassing the glial cells without provoking unspecific reactive gliosis and promote intimate contact with RGCs. This is exactly what our CNT electrodes appear to achieve, allowing us to conclude, based on our electrophysiological, structural and immunological data, that CNT electrodes offer a promising alternative for the design of new generation epi-retinal prosthetics.

## 5 Conclusions

In this study, we have maintained dystrophic retinas for up to three days *in vitro* on CNT and TiN electrodes, demonstrating for the first time that CNTs become gradually incorporated within the ILM without promoting reactive glial responses, resulting in gradual decrease in stimulation thresholds and increase in the number of RGCs reaching firing threshold. Although previous studies have already demonstrated that CNTs offer great potential for epi-retinal prosthetic devices [16], [50], [51], here we provide the first comprehensive evaluation of how CNTs interact with RGCs in the diseased retina, comprising ultrastructural, immunohistochemical and electrophysiological evidence, demonstrating a clear correlation between temporal changes in structure and function underlying the ability of CNT electrodes to stimulate RGCs and how it improves with time. Collectively, our data indicate that CNTs are indeed a promising electrode material for retinal prosthetics, with superior biocompatibility and electrical properties.

## Figures and Tables

**Figure:**
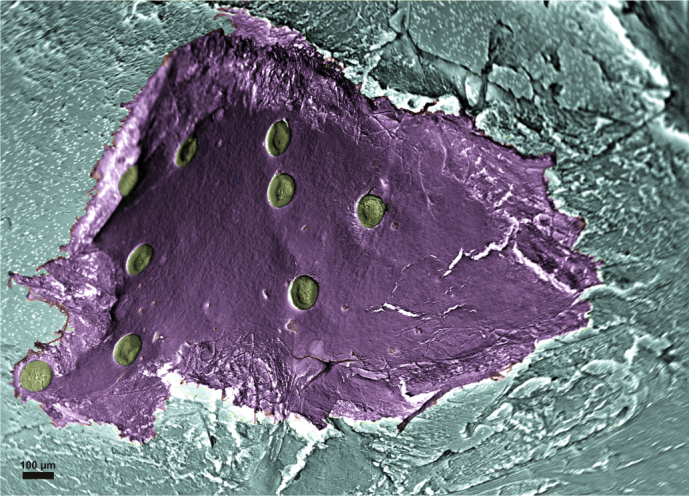


**Table 1:** Comparison of performance between different studies using electrical retinal stimulation.

Reference	Experimental setup	Species	Electrode placement	Electrode material	Electrode diameter/spacing (μm)	Pulse Width (μs)	Threshold charge density (mC/cm^2^)
57	*In vitro*	Rabbit	Epi-retinal healthy	Pt-Ir	3.56/Na	100	5–32.14
7	*In vivo*	Human	Epi-retinal dystrophic	Pt Grey	200/502.5d	450	0.29
This study	*In vitro*	Crx−/−mouse	Epi-retinal Dystrophic	TiN	30/200	10–100	0.27
This study	*In vitro*	Crx−/−mouse	Epi-retinal dystrophic	CNT	30/200	10–100	0.22
58	*In vivo*	Human	Sub-retinal dystrophic	TiN	112.83/280h 396d	2000–3000	0.2–0.6
59	*In vitro*	Mouse rd1	Epi-retinal dystrophic	TiN	30/200	100–1000	0.235–0.353
60	*In vitro*	Rat/RCS rat	Sub-retinal dystrophic	IrO_2_	60, 40, 20/285, 145, 75	4000	0.26–0.32
61	*In vitro*	Rat	Epi-retinal healthy	Pt	7-16/60	50/100	0.058
62	*In vivo*	Human	Epi-retinal healthy	Pt	500 and 250/800	1000	0.05–0.57
63	*In vitro*	Rhesus monkey	Epi-retinal healthy	Pt	9-15/60	50/100	0.05
61	*In vitro*	Rat P23H	Epi-retinal dystrophic	Pt	7-16/60	50/100	0.049
51	*In vitro*	Rat	Epi-retinal healthy	PEDOT-CNT	30/200	200	0.0032–0.0159
51	*In vitro*	Rat	Epi-retinal healthy	TiN	30/200	200	0.0159–0.0318

Thresholds are highest *in vivo* and for sub-retinal electrodes. Underlined numbers were derived from data provided in the referenced papers. Electrode spacing, h: horizontal, d: diagonal.
