# Supplementary material for: Carbon nanotube electrodes for retinal implants: A study of structural and functional integration over time
Source: Biomaterials. 2017 Jan;112:108–21. doi: 10.1016/j.biomaterials.2016.10.018 (PMC5123641; doi:10.1016/j.biomaterials.2016.10.018)
Supplement: Supplementary file 1 [file mmc1.pdf]

Supplementary Data

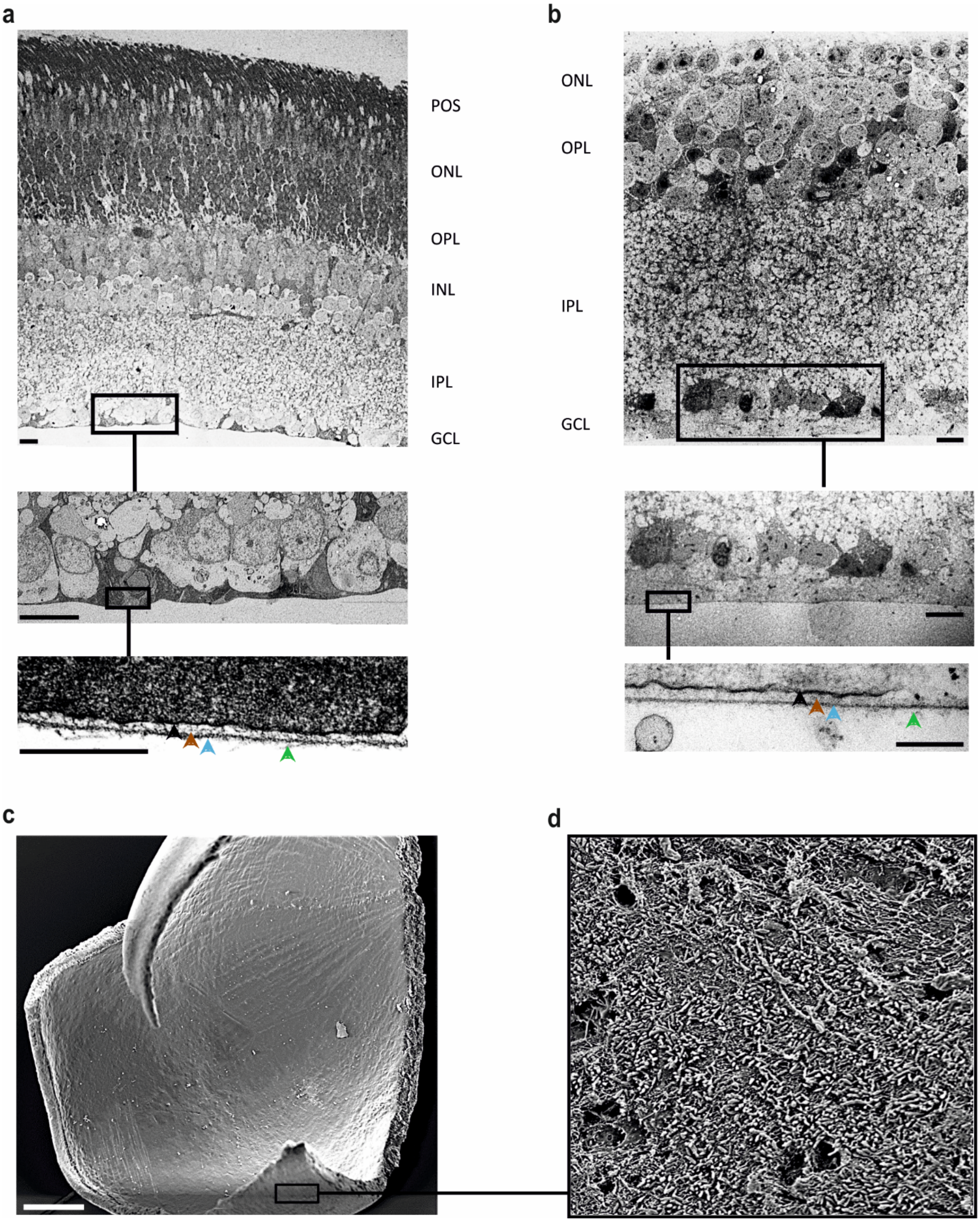

Supplementary Figure 1. Anatomical evidence of retinal degeneration in the Crx KO retina. (a-b) Transmission Electron Microscopy (TEM) micrographs depicting structure in wild type **a**) and Crx-/- (**b**) retinas with specific focus on the RGC layer and ILM. All three layers of the ILM are retained in the Crx model, with cytoplasmic membrane of a Müller cell (black arrow head); *lamina rara interna* (brown arrow head); *lamina densa* (blue arrow head); *lamina rara externa*

(green arrow head). Photoreceptor outer segments (POS), outer nuclear layer (ONL), outer plexiform layer (OPL), inner limiting membrane (ILM), inner nuclear layer (INL), inner plexiform layer (IPL), ganglion cell layer (GCL). Scale bars are 10 µm in the top two micrographs and 500 nm in the bottom micrographs. **(c)** SEM of half a Crx retina with the ILM facing upwards. **(d)** Higher magnification of black box in **(c)** showing atrophied photoreceptor outer segments on the outer retinal surface. Scale bars are 200 and 10 µm for **(c)** and **(d)**, respectively.

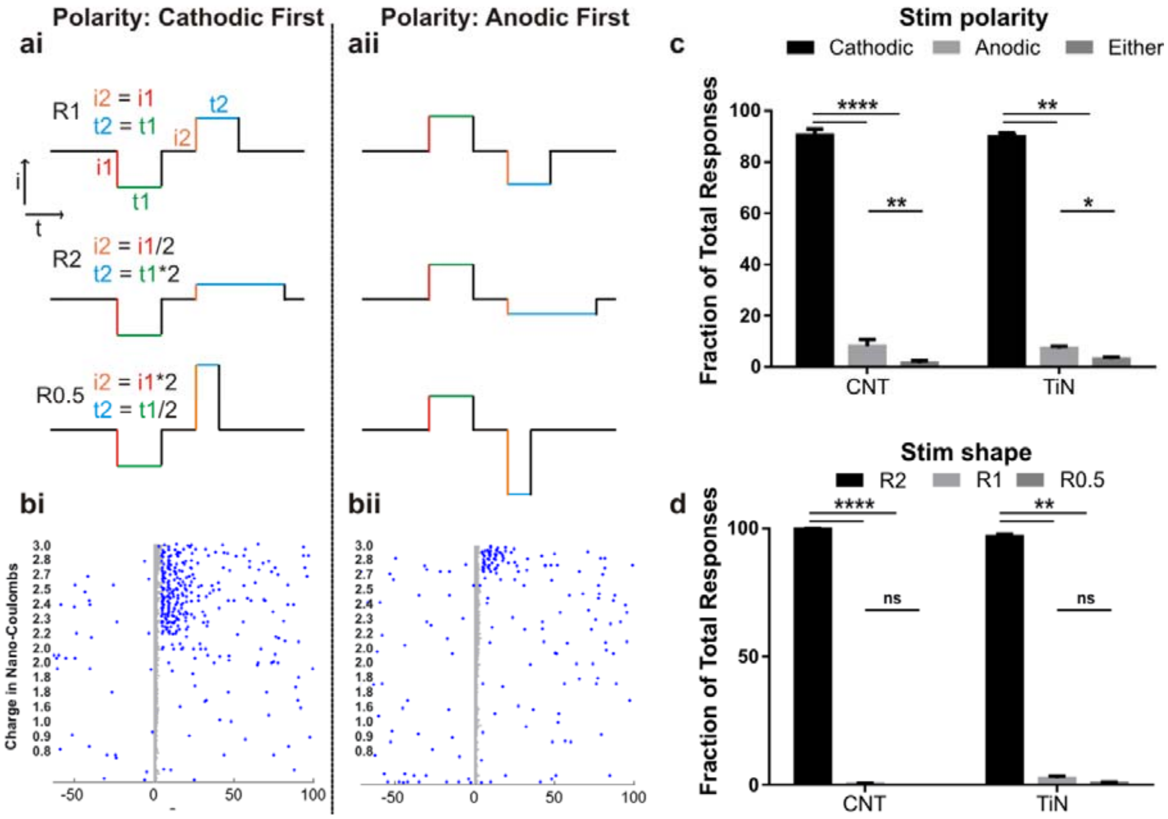

Supplementary Figure 2. Impact of stimulus polarity and shape on response threshold. (a) Different shapes of asymmetric charge balanced stimuli satisfying the relationship i1 * t1 = i2 * t2 with i and t representing current and time, respectively. Stimuli were delivered either with an initial negative (**ai**, Cathodic first) or positive (**aii** Anodic first) phase. **(b)** example rasters demonstrating a lower response threshold when the stimulus was presented with a Cathodic first stimulus **(bi)** compared to an Anodic first stimulus **(bii)**. Epochs are organised by increasing amount of stimulation charge value along the ordinate axes. These are not linear or unique as they represent the product of multiple parameters for stimulus current and single-phase duration (see methods). **(c)** Average fraction of responses with a lower threshold depending on stimulus initial polarity. Mann Whitney test, Asterisks indicate significance, $p<0.0001$, $p=0.0069$, $p=0.0022$ and $p = 0.0152$; from left to right. **(d)** Average fraction of responses with a lower threshold depending on stimulus shape. Mann Whitney test, Asterisks indicate significance, $p<0.0001$ $p>0.9999$, $p=0.0022$ and $p = 0.2100$; from left to right.

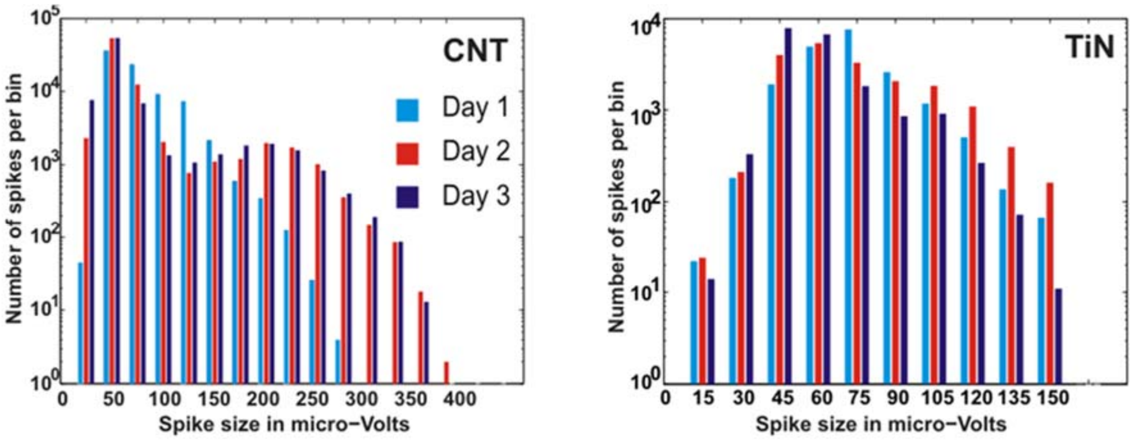

Supplementary Figure 3. Acute increase in spike amplitudes. Histogram distribution of spike amplitudes as a function of time interfacing on a CNT (left) or TiN (right) array.

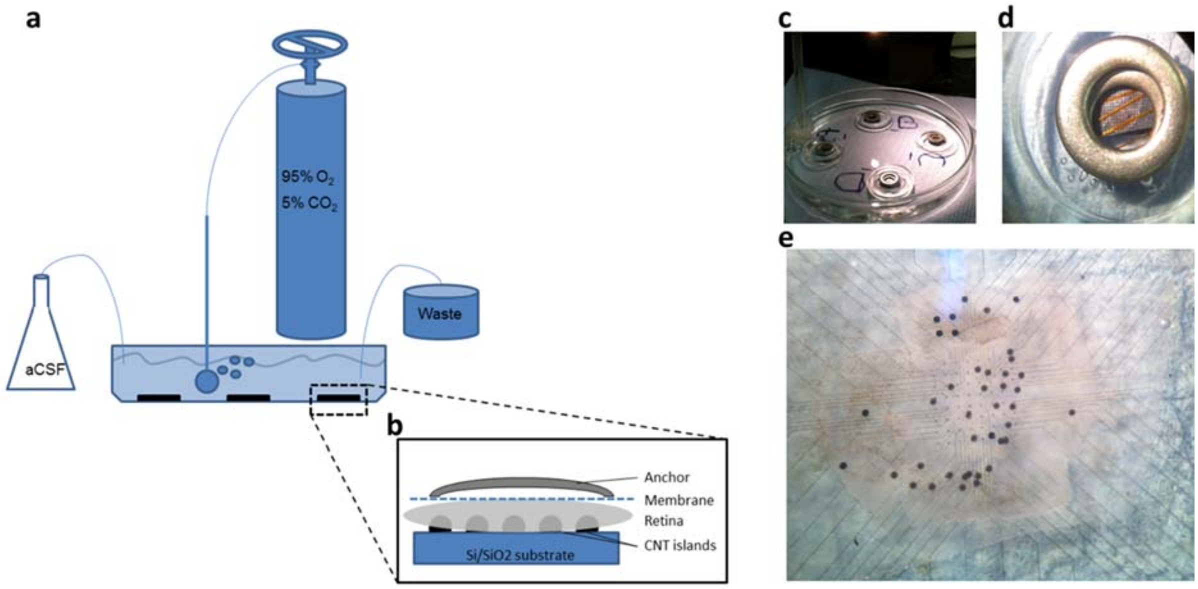

Supplementary Figure 4. Generating viable CNT-Retina biohybrids. (a) Schematic diagram depicting the incubation chamber, in which biohybrids were maintained under physiological conditions for up to 72 hours. **(b)** diagram showing the various layers in an individual bio-hybrid retina. **(c)** Photograph of an incubation chamber. **(d)** Overhead photograph of a biohybrid during incubation, as depicted in the insert for panel **(a). (e)** Retina-CNT biohybrids, with multiple large CNT islands attached to the GCL (peeled off their substrate by the Velcro effect between CNTs and retinal tissue), being tested for viability on a MEA.

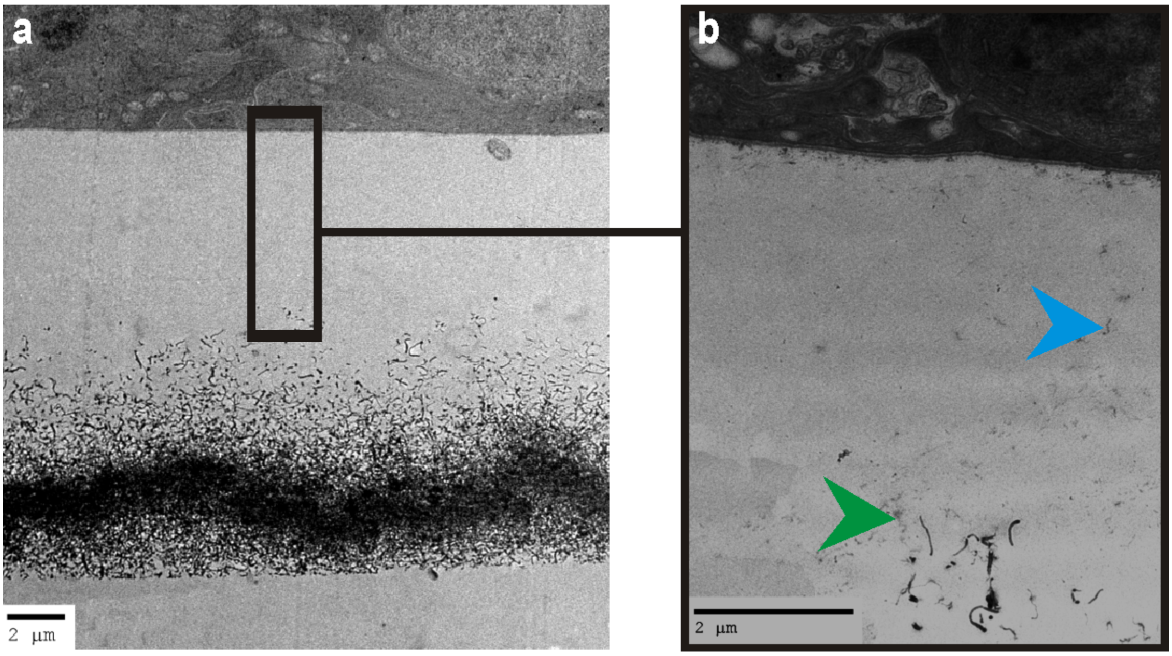

Supplementary Figure 5. CNTs 2 μm away from retina after 4 hours. (a) Low magnification TEM of the CNT-retina interface. **(b)** High magnification of black box in **(a)**, with individual CNTs (blue arrowhead) 2 μm away from the ILM and fibrils from the vitreous (green arrowhead) in contact with CNTs.

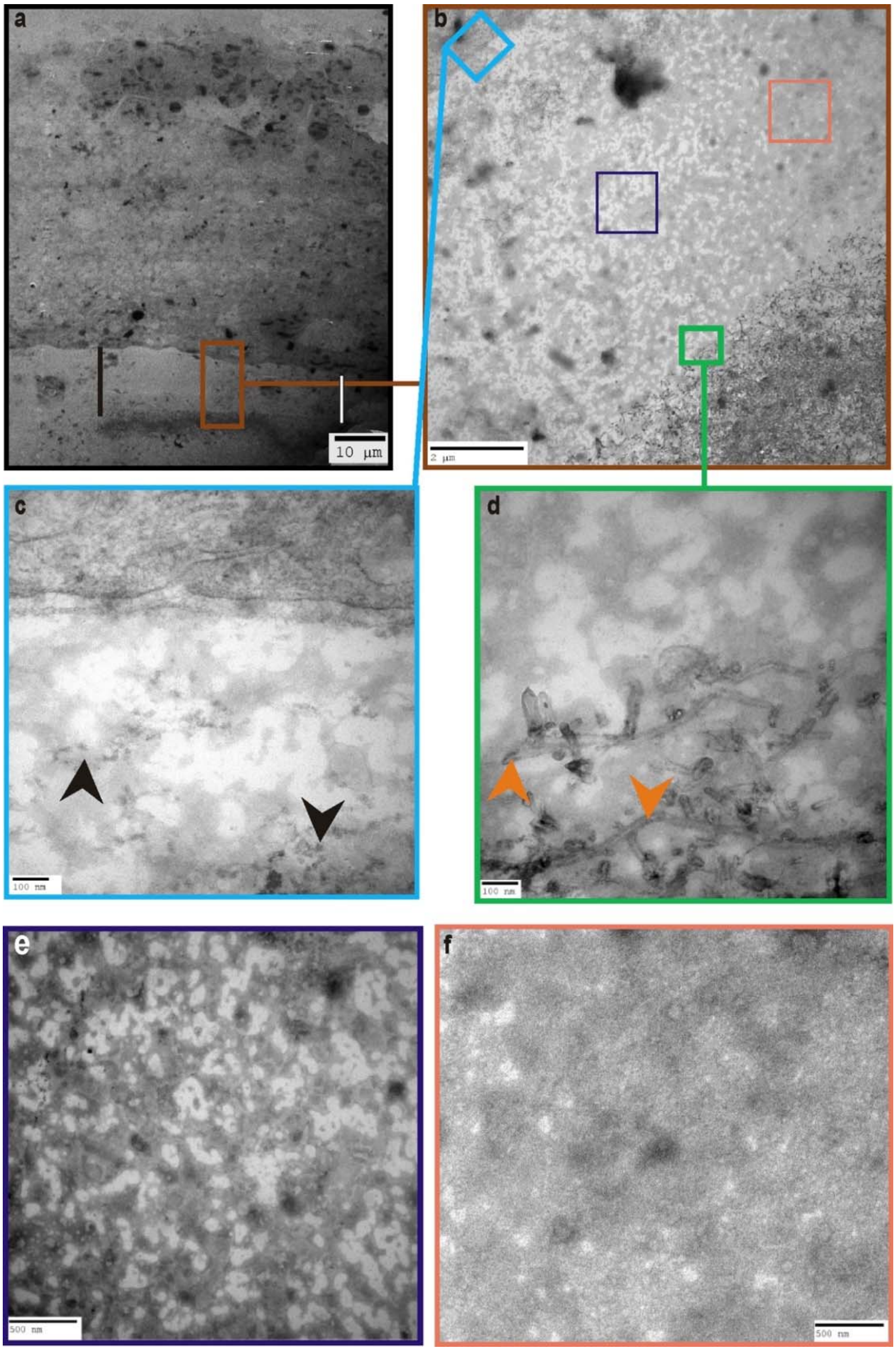

Supplementary Figure 6. Thickening of vitreous as CNT island reaches closer to the retina after 12 hours. (a) the edge of the island is farther away from the retina (black line, 15.6 μm) than the part close to the centre (white line, 9.9 μm). **(b)** Magnified image of the brown box in **(a). (c)** Magnified image of the blue box in **(b)** detailing the edge of the ILM with thick protein strands (black arrowheads). **(d)** High magnification of green box in **(b)**, with individual CNTs (orange arrows). **(e)** high magnification of purple box in **(b)**, showing a denser vitreal matrix than in **(c)** and **(d). (f)** High magnification of orange box in **(b)** which shows a denser matrix than in **(e)**.

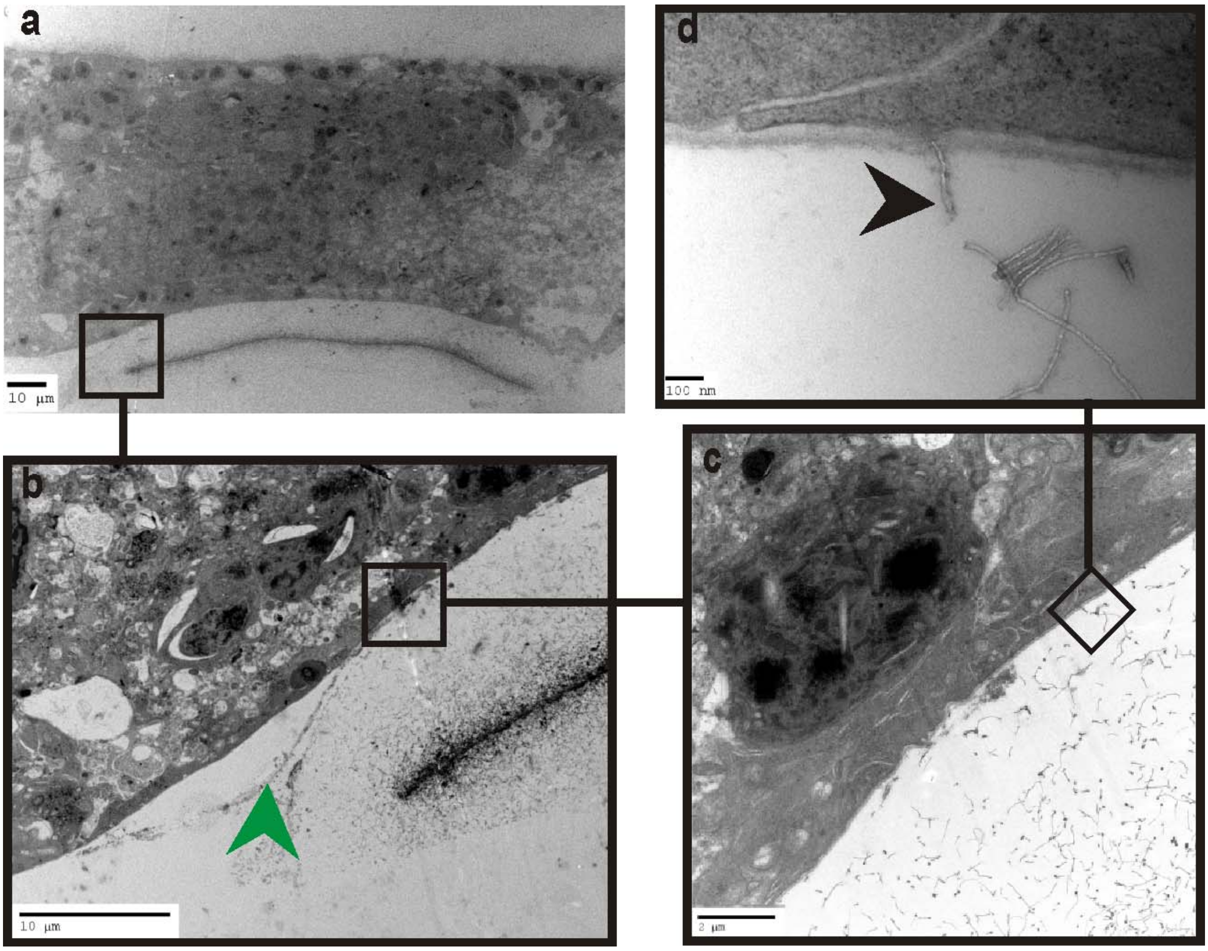

Supplementary Figure 7. Formation of accessory limiting membrane after 24 hours: (a) Low magnification TEM of retina bending to accommodate CNT island. **(b)** Higher magnification of black box in **(a)**, focusing on the edge of the CNT island and imaging the formation of an accessory limiting membrane (green arrowhead). **(c)** Higher magnification of black box in **(b)**, focusing on the ILM. **(d)** Higher magnification of black box in **(c)**, focusing on an individual CNT (black arrowhead) penetrating the ILM.

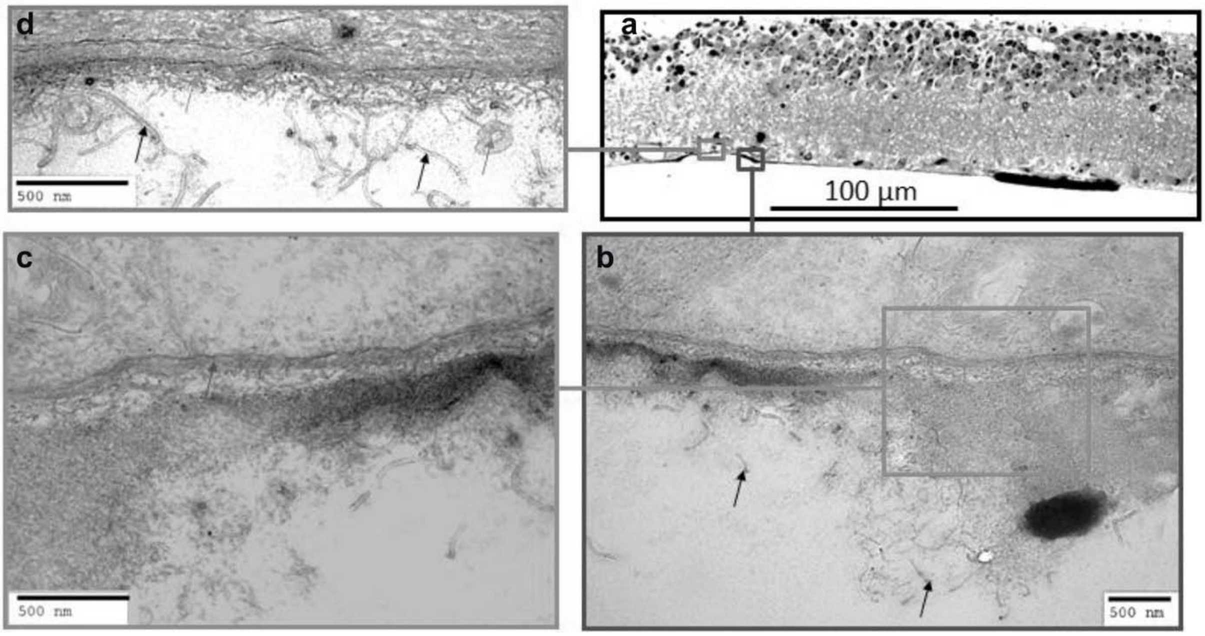

Supplementary Figure 8. CNTs embedded in matrix following the ripping of island from ILM after 48 hours. (a) light micrograph of a semi-thin section of P90 Crx -/- retina interfaced with a CNT island for 48 hours. **(b-d)** increasingly high magnification TEM micrographs of ultra-thin sections of the same retina focusing on the ILM, with the CNTs embedded in retinal secretion. The CNT island's apex is displayed in **(d)**, where individual CNTs are seen adhering to vesicular elements and collagen fibrils.

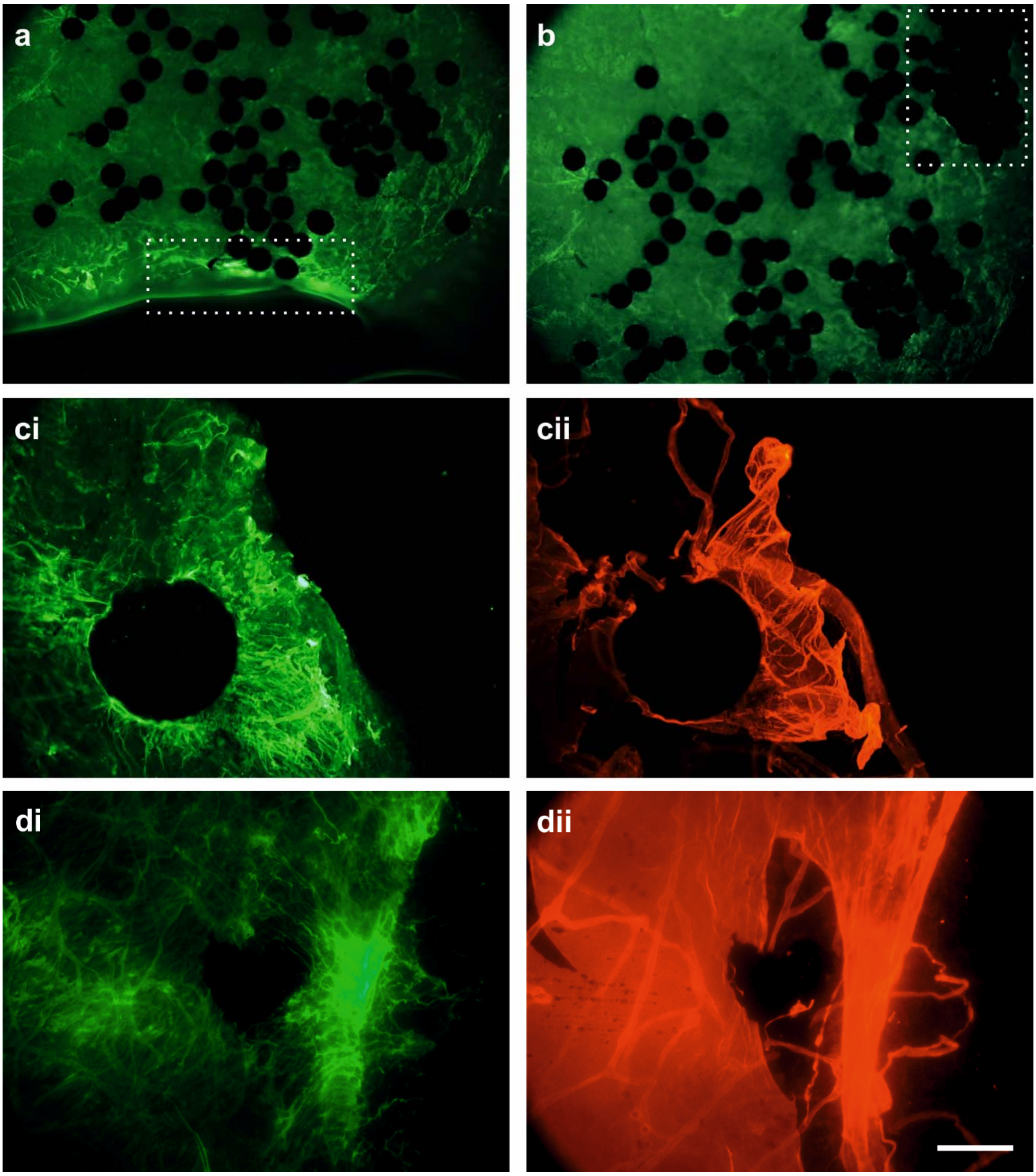

Supplementary Figure 9. CNT islands embedded in retina biohybrids. Wholemount micrographs of 30 µm **(a-b)** and 100 µm **(c-d)** CNT islands embedded into the RGC layer side of retina biohybrids stained with GFAP **(a, b, ci, di)** and laminin **(cii, dii).** **(a)** Very few 30 µm islands induced up-regulation of GFAP (brighter fluorescence in white dotted rectangle). **(b)** Some clusters of CNT islands were pulled together (white dotted rectangle) as observed in SEM micrographs of Figure 6b. 100 µm CNT islands induced high levels of gliosis **(ci, di)** as the generally smooth structure of the ILM's laminin is completely disrupted **(cii, dii)**. Scale bar represents 50 µm for **(a-c)** and 70 µm for **(d)**.
